# Transcriptional analysis reveals the metabolic state of *Burkholderia zhejiangensis* CEIB S4-3 during methyl parathion degradation

**DOI:** 10.7717/peerj.6822

**Published:** 2019-04-24

**Authors:** María Luisa Castrejón-Godínez, Ma. Laura Ortiz-Hernández, Emmanuel Salazar, Sergio Encarnación, Patricia Mussali-Galante, Efraín Tovar-Sánchez, Enrique Sánchez-Salinas, Alexis Rodríguez

**Affiliations:** 1 Centro de Investigación en Biotecnología, Universidad Autónoma del Estado de Morelos, Cuernavaca, Morelos, Mexico; 2 Centro de Ciencias Genómicas, Universidad Nacional Autónoma de México, Cuernavaca, Morelos, Mexico; 3 Centro de Investigación en Biodiversidad y Conservación, Universidad Autónoma del Estado de Morelos, Cuernavaca, Morelos, Mexico

**Keywords:** Gene expression, Pesticide biodegradation, Methyl parathion, Transcriptomic analysis

## Abstract

*Burkholderia zhejiangensis* CEIB S4-3 has the ability to degrade methyl parathion (MP) and its main hydrolysis byproduct *p*-nitrophenol (PNP). According to genomic data, several genes related with metabolism of MP and PNP were identified in this strain. However, the metabolic state of the strain during the MP degradation has not been evaluated. In the present study, we analyzed gene expression changes during MP hydrolysis and PNP degradation through a transcriptomic approach. The transcriptional analysis revealed differential changes in the expression of genes involved in important cellular processes, such as energy production and conversion, transcription, amino acid transport and metabolism, translation, ribosomal structure and biogenesis, among others. Transcriptomic data also exhibited the overexpression of both PNP-catabolic gene clusters (*pnpABA′E1E2FDC* and *pnpE1E2FDC*) present in the strain. We found and validated by quantitative reverse transcription polymerase chain reaction the expression of the *methyl parathion degrading* gene, as well as the genes responsible for PNP degradation contained in two clusters. This proves the MP degradation pathway by the strain tested in this work. The exposure to PNP activates, in the first instance, the expression of the transcriptional regulators multiple antibiotic resistance regulator and Isocitrate Lyase Regulator (IclR), which are important in the regulation of genes from aromatic compound catabolism, as well as the expression of genes that encode transporters, permeases, efflux pumps, and porins related to the resistance to multidrugs and other xenobiotics. In the presence of the pesticide, 997 differentially expressed genes grouped in 104 metabolic pathways were observed. This report is the first to describe the transcriptomic analysis of a strain of *B. zhejiangensis* during the biodegradation of PNP.

## Introduction

Organophosphorus pesticides are one of the most commonly used groups of pesticides worldwide (Tiwari et al., 2018); they have been used extensively in agricultural and household pest control, mainly against insects (Pope, 1999). Their intensive use is often related to environmental pollution events in agricultural soils and the surrounding water (Diagne, Oturan & Oturan, 2007; Begum & Arundhati, 2016; Ahn et al., 2018; Tiwari et al., 2018). These pesticides can inhibit the activity of the acetylcholinesterase enzyme (AChE, EC 3.1.1.7), resulting in the accumulation of the neurotransmitter acetylcholine in synapses. The increase in the acetylcholine concentration causes overstimulation of muscles, glands and the nervous system, causing convulsions, paralysis, and eventually death of insects and mammals (Van Dyk & Pletschke, 2011; Pailan et al., 2015; Briceño et al., 2016). Methyl parathion (MP) is an organophosphate pesticide that is broadly used in agriculture (Moreno-Medina, Sánchez-Salinas & Ortiz-Hernández, 2014) and has been classified by the World Health Organization as an extremely hazardous compound (Azaroff, 1999; Diagne, Oturan & Oturan, 2007). This organophosphorus pesticide is susceptible to microbial degradation, through a process mediated by hydrolytic enzymes, which releases *p*-nitrophenol (PNP) as the main product (Wang et al., 2014). This compound has several industrial applications, mainly as a precursor in the synthesis of drugs, dyes, explosives, herbicides, and fungicides, among others (Levin et al., 2016). However, in the environment, MP is considered to be a highly toxic pollutant, especially for soil microbiota (Min, Wang & Hu, 2017). Different microorganisms have been reported to have the capability to hydrolyze MP (Zhao et al., 2014); however, the number of microorganisms with metabolic strategies for the biodegradation of both MP and PNP is still limited.

Previously, information about the strain *Burkholderia zhejiangensis CEIB* S4-3, which was isolated from agricultural soils from the state of Morelos, in central Mexico, was reported. This strain was found to have the ability to hydrolyze MP, to degrade PNP and to use the byproducts as a carbon source when cultured in the presence of 0.2 mM of MP in liquid fermentation (Popoca-Ursino et al., 2017). The strain *B. zhejiangensis* CEIB S4-3 is able to hydrolyze MP pesticide in 1 h and completely degrade PNP in 12 h. The analysis of a draft genome of this strain (Hernández-Mendoza et al., 2014) revealed the presence of several genes that encode sequences that are highly similar to previously characterized enzymes related to the hydrolysis of MP and the degradation of PNP in *Burkholderia* sp. (Vikram et al., 2012) and *Pseudomonas* sp. (Zhang et al., 2012). The genomic data revealed that *B. zhejiangensis* CEIB S4-3 has a methyl parathion degrading (*mpd*) gene with 99% identity with respect to the gene MpdB, which was identified in *B. cepacia* by Ekkhunnatham et al. (2012), which codes for a methyl parathion hydrolase (MPH) enzyme. Furthermore, this data also reveals that this strain has two PNP catabolic gene clusters, *pnpABA′E1E2FDC* and *pnpE1E2FDC* (Hernández-Mendoza et al., 2014; Popoca-Ursino et al., 2017). The alignment between the two PNP proteins of *B. zhejiangensis* CEIB S4-3 and *Burkholderia* sp. SJ98 showed an identity of 67% and 100%, respectively; a similarity of 79% and 100%, respectively; and an *E*-value of 3*e*^−71^ and 0.0, respectively (Popoca-Ursino et al., 2017; Vikram et al., 2012, 2013).

Recently, modern technologies, such as genomics, transcriptomics, proteomics, and metabolomics, have been applied to the study of a sole microorganism or microbial communities implicated in pollutant bioremediation processes (Chakraborty, Wu & Hazen, 2012). The scientific information generated through these approaches makes it possible to obtain a better understanding of the key factors that are involved in the biodegradation and biotransformation of xenobiotics, as well the mechanisms to fight the cell stress induced by these chemicals (De Lorenzo, 2008; Dvořák et al., 2017; Pratap et al., 2017).

Transcription is an essential step in the expression of genes, and understanding this process is of great interest for molecular and cellular biology in several research areas. In the field of environmental and bioremediation sciences, transcriptomics is a useful technique for obtaining a better understanding of the microbial expression and regulation of genes in response to the presence of different xenobiotic compounds, including pesticides. Through transcriptomic analysis, it is possible to explore the molecular mechanisms related to pesticide bacterial degradation and to use this knowledge for the development of pesticide bioremediation approaches. The transcriptional changes during the bacterial biodegradation of several pesticides, such as 2,4-dichlorophenoxyacetic acid, ortho-phenylphenol, glyphosphate, and paraquat, have been studied through different methodologies, such as microarrays, total transcript amplification, quantitative reverse transcription polymerase chain reaction (qRT-PCR), and RNA-Seq (Dennis et al., 2003; Nde et al., 2008; Jang et al., 2008; Allen & Griffiths, 2012; Huang et al., 2013; Luo et al., 2014; Li et al., 2015; Namouchi et al., 2016).

Knowledge of the profiles and abundance of RNA species is a key factor in understanding the biology and physiology of cells under a specific condition, and the entire set of RNA molecules is denominated as a transcriptome (Dong & Chen, 2013). Since the 1990s, several technologies have been developed for study of the transcriptome, from the serial analysis of gene expression to microarrays, and in the most recent years, RNA-Seq (McGettigan, 2013; Lowe et al., 2017). At present, RNA-Seq is the next generation technology for high-throughput RNA sequencing; it gives a detailed picture about the transcriptional profile of a biological sample (Wang, Gerstein & Snyder, 2009; Young et al., 2010; Hrdlickova, Toloue & Tian, 2017), even from a single bacterial cell (Wang et al., 2015). RNA-Seq data are commonly used to define the genome-wide transcript expression profiling and the identification of differentially expressed genes (DEGs) among different biological or experimental conditions (Han et al., 2015; Pereira, Imada & Guedes, 2017). RNA-Seq has emerged as a powerful tool for transcriptomic analysis in the field of environmental microbiology, which has especially been applied in the assessment of the microbial diversity in polluted sites (Yan et al., 2017), as well as in the discovery and characterization of genes implicated in the tolerance, biodegradation, and bioremediation of xenobiotics by different microorganisms (Zhang et al., 2015; Yang et al., 2016; Albers et al., 2018).

Several authors reported pesticide transcriptomic studies on microorganisms, such as *Pseudomonas aeruginosa*, *Staphylococcus aureus*, *B. thailandensis*, *Escherichia coli* O157:H7, *Penicillium digitatum,* and *Trichoderma atroviride* T23 (Dennis et al., 2003; Nde et al., 2008; Jang et al., 2008; Kang et al., 2011; Allen & Griffiths, 2012; Liu et al., 2015; Zhang et al., 2015). Through these studies, overexpressed genes that encode enzymes related to the biodegradation of these types of xenobiotics, the catabolic pathways implicated in their use as a carbon source and for energy generation, the negative effects on microbial metabolism because of pesticide exposure, and the genetic strategies employed by these microorganisms to minimize these effects, were identified.

The aim of this study was to analyze the transcriptional changes experienced by the *B. zhejiangensis* strain CEIB S4-3 in response to MP presence as a tool to identify the expressed genes related to its biodegradation. Furthermore, understanding the changes in the gene expression profile will assist in understanding the mechanisms that are used by the cell to contend with the stresses caused both by this pesticide and by the absence of an additional carbon source.

## Materials and Methods

**Figure 1:**
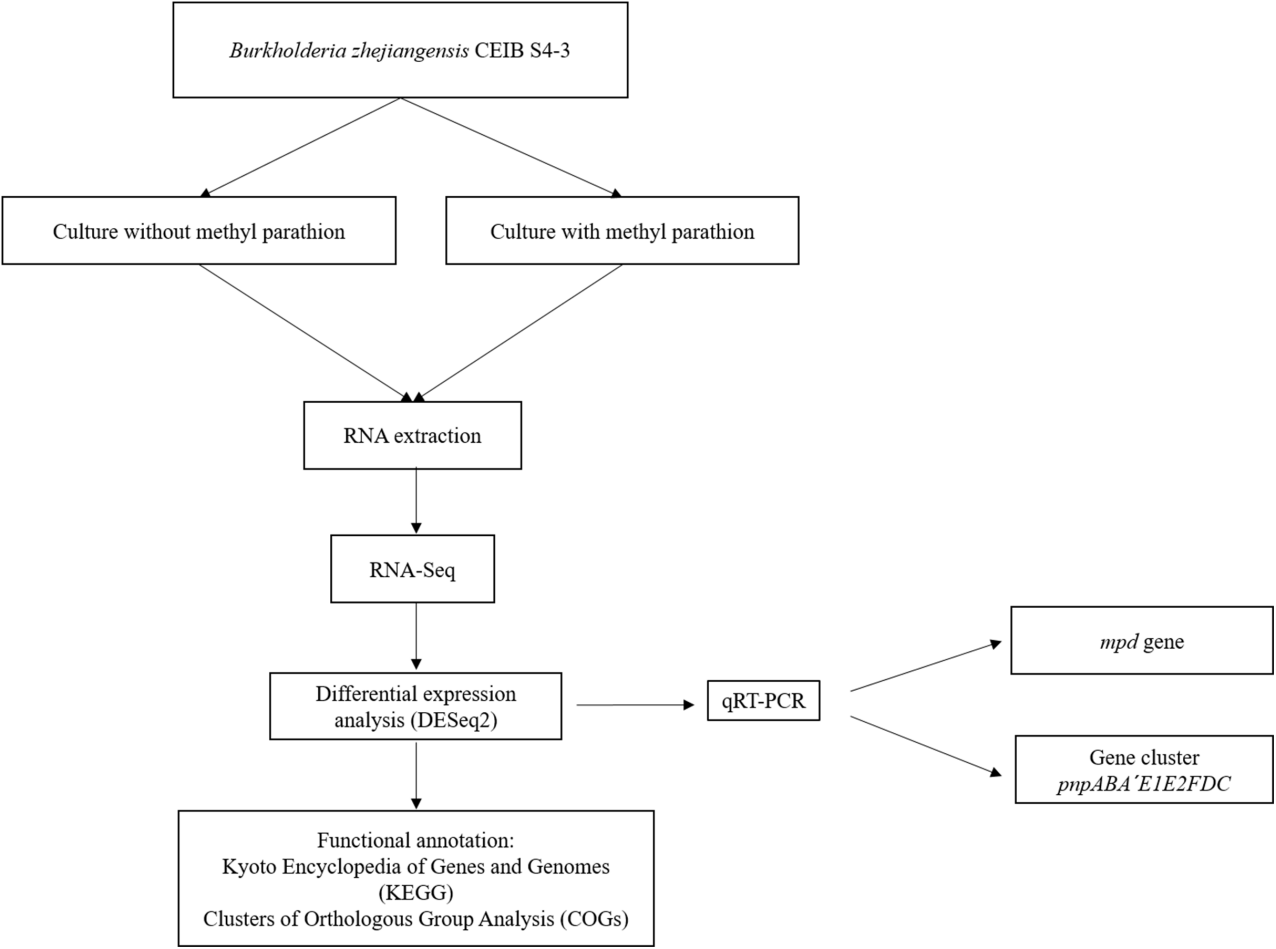
Flow diagram of the general experimental strategy of the study. The diagram illustrates the sequence of experiments and the bioinformatic analysis carried out in this work.

Figure 1 shows the flow diagram of the experimental strategy used in this study.

### Pesticide

Analytical grade MP (*O,O-dimethyl O-4-nitrophenyl phosphorotioate*) with 98% purity was purchased from Ultra Scientific, Analytical Standards, USA. A stock of MP (38 mM) was prepared using HPLC-grade methanol as a solvent.

### Bacterial strain and culture conditions

The culture conditions were carried out according to those reported by Popoca-Ursino et al. (2017). To obtain biomass for the inoculum, tryptone soy broth (Bioxon, Becton Dickinson, Mexico State, Mexico) and tryptone soy agar (Bioxon, Becton Dickinson, Mexico State, Mexico) were used. For the MP hydrolysis by *B. zhejiangensis CEIB* S4-3, the cells were inoculated in to minimal medium (MM), with the following composition per liter: KH_2_PO_4_, 0.82 g; K_2_HPO_4_, 0.19 g; MgSO_4_ 7H_2_O, 0.20 g; KNO_3_, 2 g; (NH_4_)_2_SO_4_, 0.99 g (Solution A). In addition, two ml of solution B was added per liter of solution. The solution B composition was as follows (per liter): H_3_BO_3_, 2.8 g; MnSO_4_ H_2_O, 2.55 g; CuSO_4_ 5H_2_O, 0.17 g; CoCl_2_ 6H_2_O, 2.43 g; ZnSO_4_ 7H_2_O, 0.25 g. All the salts of the B solution were sterilized separately.

### MP hydrolysis and analytical methods

*Burkholderia zhejiangensis* CEIB S4-3 was cultivated in 250 ml Erlenmeyer flasks with 50 ml of MM previously sterilized at 121 °C for 15 min. Nine flasks with MM were supplemented with MP to a final concentration of 0.2 mM. The same number of experiments were carried out without pesticide, which were considered to be the control. Concentrations over 0.4 mM showed a toxic effect on bacterial cells, decreasing the viability of the culture significantly (Popoca-Ursino et al., 2017), and was the reason why the medium was not supplemented with higher concentrations of MP. All flasks were incubated at 30 °C with constant shaking at 150 rpm. The cell density in the culture was measured at 600 nm using BioMate 3 of the Thermo Scientific spectrophotometer, and three biological replicates were performed. PNP concentration released by MP hydrolysis was measured through UV–Vis spectroscopy at 410 nm and was calculated according to a calibration curve of absorbance vs concentration 0.01–0.36 mM of PNP.

In order to examine hydrolysis of MP, three sampling times points were selected for analysis of the experimental treatments containing 0.2 mM MP. The first at the beginning of the experiment (0 h); the second, after 3 h, when the PNP concentration reached a higher level as a result of the hydrolysis of MP by the bacterial culture; and, finally, the third sampling time was after 9 h, when the PNP concentration decreased. In the experimental treatment without the presence of the pesticide, the same sampling times were used. Bacterial biomass for both experimental treatments were collected by centrifugation, according to the sampling times 0, 3, and 9 h; the recovered biomasses were frozen in liquid nitrogen and stored at −70 °C for subsequent RNA extraction in the presence of RNAlater^®^ stabilization solution (Ambion, Foster City, CA, USA).

### RNA extraction

Total RNA extraction from each sampling point was carried out according to the protocol of the TRI Reagent® Kit. Subsequently, for the elimination of the DNA remains, the samples were treated with five µl of Thermo Scientific DNAse I; and finally, for its purification, the “RNA Clean and Concentrator” columns of the Zymo Research brand were used. The concentration of RNA was determined by measuring its absorbance using the Nanodrop 200c spectrophotometer (Thermo Scientific, Waltham, MA, USA). The RNA integrity number value was 9.0 on average.

### RNA-Seq

After RNA extraction, rRNA was eliminated using the RiboZero Kit (Illumina, San Diego, CA, USA), and then the depleted RNA was used for cDNA library construction. Sequencing was performed by Illumina HiSeq 2 × 150 bp by GENEWIZ Company. The RNA-Seq readings were processed using different software. FastQC was used to determine the quality of the sequences and, for the trimming, the CLC Genomics Workbench adapter trimming and quality trimming were used. The minimum read length after trimming should be 35 bp, and any sequence shorter than 35 bp was discarded and not considered in the analysis.

The processed sequences were aligned with the draft genome of *B. zhejiangensis* CEIB S4-3 as a reference genome using the CLC Genomics Workbench Server v. 10.0.1 to infer the overexpression of transcripts according to Thorvaldsdóttir, Robinson & Mesirov (2013). The draft genome sequence of *B. zhejiangensis* CEIB S4-3 (including 154 scaffolds) has been deposited in GenBank database under accession no. JSBM00000000 (Hernández-Mendoza et al., 2014). A mismatch cost 2, insertion cost 2, deletion cost 3, and similarity fraction 0.8 were used. We extracted counts from the above mapping and ran DESeq2 on those counts. We used custom scripts to perform the analysis. Finally, for the analysis of expression profiles, DESeq2 was employed (Bech et al., 2017; Snoeck et al., 2017; Blunder et al., 2018).

### Analysis of functional annotation of genes

To locate the DEGs in their corresponding metabolic pathways, an analysis of the transcripts obtained for both experimental conditions was performed in the Kyoto Encyclopedia of Genes and Genomes (KEGG) Pathway Color. The knockout (KO) genes were obtained from the Integrated Microbial Genomes & Microbiomes database (IMG/M), which can be found at the following address: https://img.jgi.doe.gov. These KOs were used in the KEGG database (http://www.genome.jp/kegg/tool/map_pathway2.html) using *Burkholderia* sp. RPE67 with the organization code “bue”.

### Clusters of orthologous group analysis

For the functional categories analysis of the clusters of orthologous groups (COGs) of proteins, the Integrated Microbial Genomes and Microbiomes (https://img.jgi.doe.gov) of the *B. zhejiangensis* CEIB S4-3 genes database were used. The overexpressed genes were classified according to a minimum fold change of ±1.5 times to a *p*-value < 0.05 (Galperin et al., 2014).

### Validation by qRT-PCR

The RevertAid First Strand cDNA synthesis kit (Thermo Scientific, Waltham, MA, USA) was used for the synthesis of cDNA from total RNA. To validate the transcriptomic data, real-time quantitative PCR was used to obtain an independent assessment of the expression of the *mpd* gene and the genes that integrate both PNP biodegradation pathways. The cDNA previously synthesized was used as a template for qRT-PCR experiments; the specific primers for each gene are presented in Table S1. Each reaction mixture contained five µl of SYBR green PCR master mixture (Thermo Scientific, Waltham, MA, USA), two µl of H_2_O, forward and reverse primers in two µl, and the template in one μl. PCRs were performed with the Rotor-Gene Q (Qiagen, Hilden, Germany) using the following program: 50 °C for 2 min and 95 °C for 10 min, followed by 40 cycles of 95 °C for 15 s, 60 °C for 30 s, and 72 °C for 30 s. The dissociation protocol was 95 °C for 15 s and 60 °C for 20 s, followed by a ramp from 60 to 95 °C for 20 min. The transcript of the recombinase A protein (*recA*) was used as an internal (unregulated) reference for relative quantification. The results of real-time RT-PCR were analyzed using the 2^−ΔΔ*C*^_T_ method (Livak & Schmittgen, 2001), and the data are expressed below as relative levels of expression. All reactions were done in triplicate (Table S1).

## Results

### MP hydrolysis activity by *B. zhejiangensis* CEIB S4-3

Based on the observed release of PNP since the beginning of the assay, *B. zhejiangensis* CEIB S4-3 carried out a fast MP hydrolysis. After 3 h, PNP reaches its maximum concentration.

**Figure 2:**
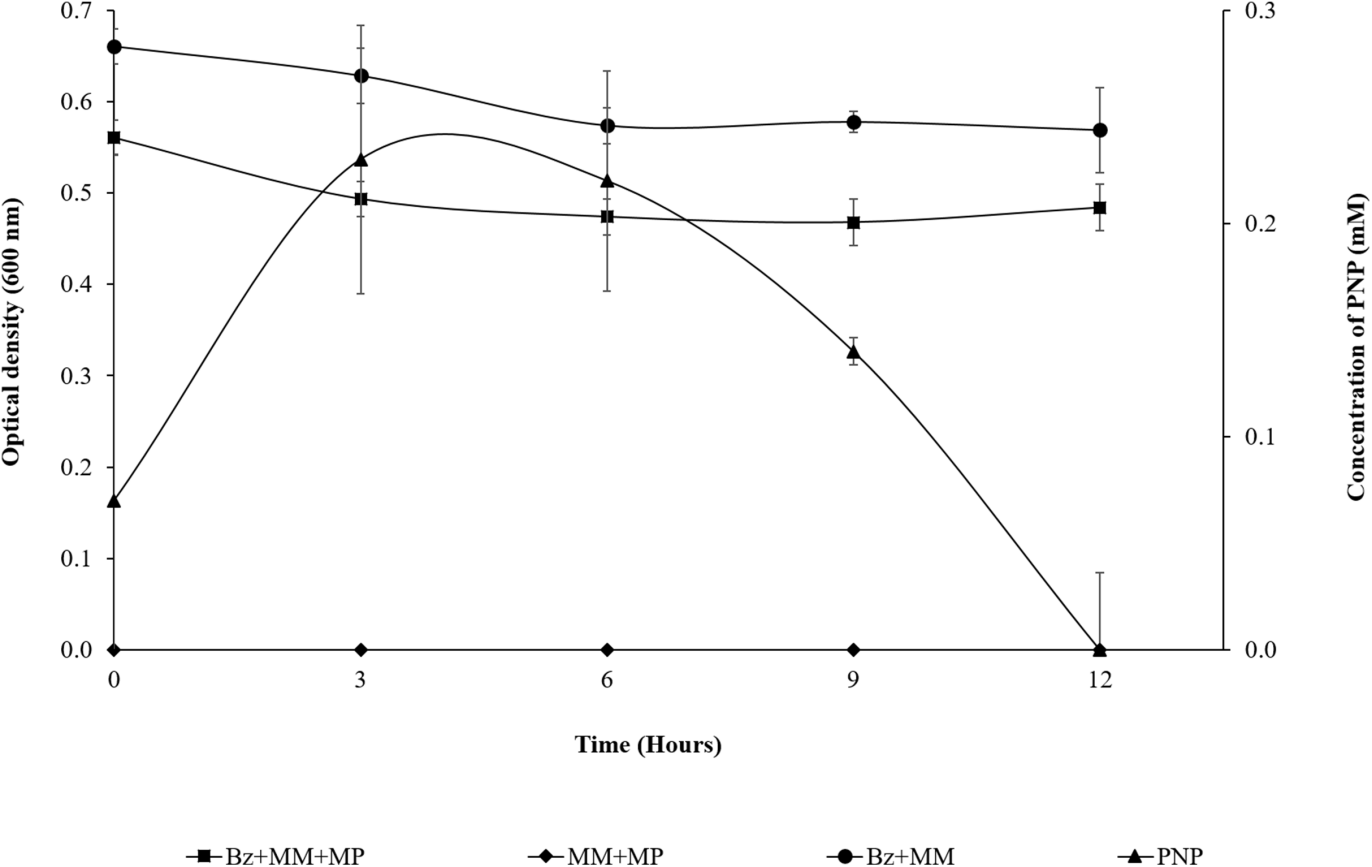
MP hydrolysis activity and PNP degradation by *Burkholderia zhejiangensis* CEIB S4-3 and its effects over the cellular population. Bz + MM + MP, Experimental condition with *B. zhejiangensis* CEIB S4-3 in minimal medium and methyl parathion at 0.2 mM. MM + MP, Experimental condition with minimal medium and methyl parathion at 0.2 mM. Bz + MM, Experimental condition with bacterial culture in minimal medium without methyl parathion. PNP, Represent the concentration of *p*-nitrophenol released from methyl parathion hydrolysis mediated by *B. zhejiangensis* CEIB S4-3. Error bars indicate standard deviation of three replicates.

As observed in Fig. 2, the presence of MP in the bacterial population at the beginning of the assay (0 h) shows a DO_600 nm_ of 0.561; in the first 3 h of the experiment, the population was slightly reduced (14%), probably due to the PNP toxicity. However, from the 3 h time point to the end of assay, the strain maintains an absorbance near 0.5. This suggested that it does not undergo growth, but the cell population is probably still viable in the culture medium at such cellular concentrations that they are able to completely degrade the pesticide within 12 h, using it as the carbon and energy source needed for cellular maintenance. Likewise, the DO_600 nm_ of the strain + MM remains in the same range over time as a result of the lack of a carbon source. The MM + MP did not present hydrolysis of the pesticide because PNP was not generated, which suggested that the MP hydrolysis and the subsequent degradation of PNP is due only to the enzymatic activity of the strain of *B. zhejiangensis* CEIB S4-3 (Fig. 2).

From the prior experiment, three sampling points were determined for the transcriptome analyses as follows: The first sampling point was at the beginning of the assay, where the presence of PNP was recorded (0.07 mM), which occurred between 5 and 10 min during the sampling time processes (a very rapid hydrolysis reaction). The second sampling point was after 3 h, where the PNP reached its maximum concentration (0.23 mM). The third sampling point was at 9 h, when the PNP concentration decreased as result of its degradation (0.14 mM) (Fig. 2).

### Gene expression analysis of *B. zhejiangensis* CEIB S4-3

As a result of the transcriptomic analysis of both conditions, with 0.2 mM MP and without the presence of the pesticide, a total of 6,941 transcripts were observed at the beginning of the experiment (0 h); 6,810 transcripts at 3 h; and finally, 6,741 transcripts were observed at 9 h. The total number of genes observed in all conditions was 7,096 protein-coding genes and 75 noncoding genes (tRNAs, ribosomal RNAs, among others). To identify the genes with a differential expression profile (DEGs) in each of the conditions, an analysis was performed using the DESeq2 program in which the fold change values were calculated for all transcripts and the genes with a differential expression profile were those that showed a fold change of at least ±1.5 (*p*-value < 0.05).

**Figure 3:**
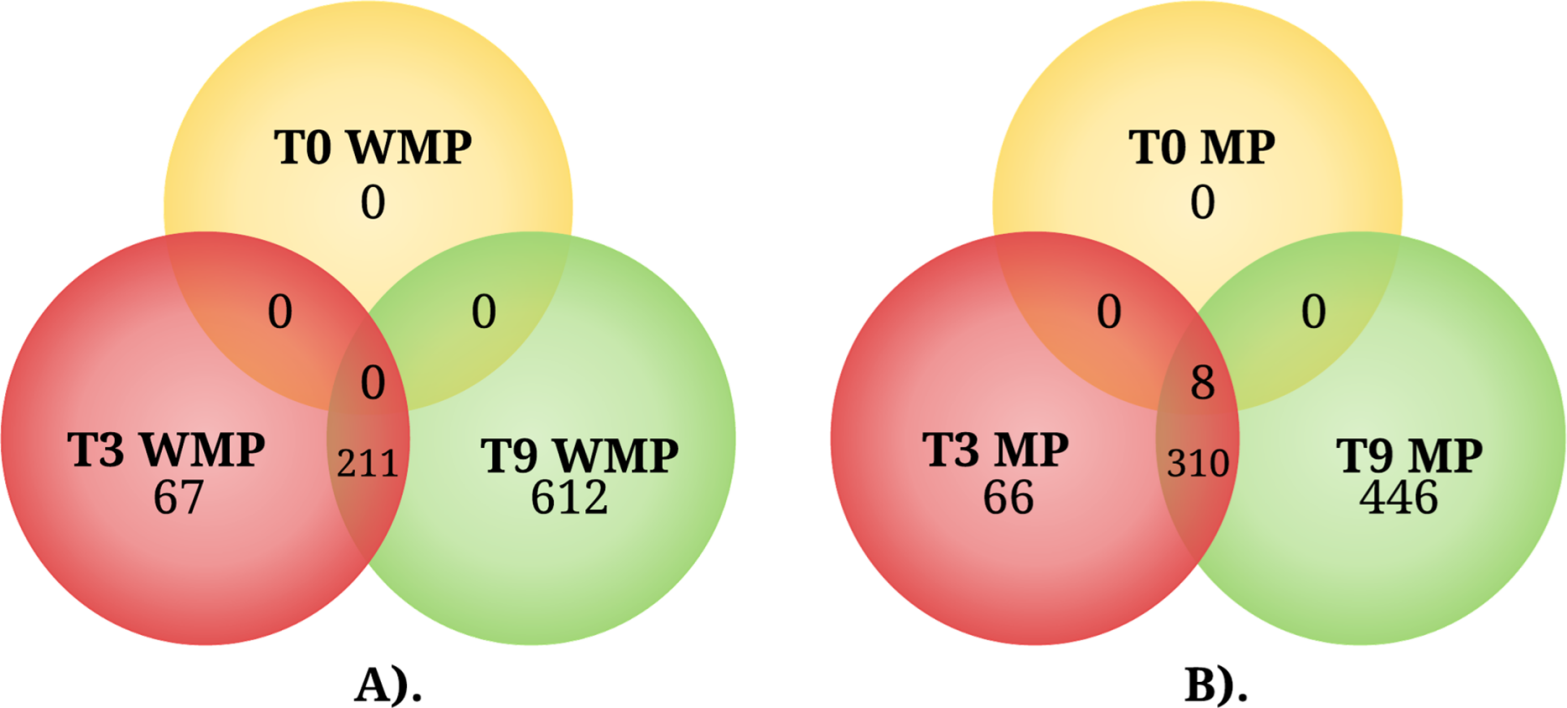
The Venn diagram represents the differentially expressed genes shared by condition among three different times. (A) Genes expressed in the absence of MP (WMP) and (B) genes expressed in the presence of MP. T0, 0 h, T3, 3 h, and T9, 9 h.

In the experimental condition without MP, the transcriptional analysis revealed that the number of DEGs increased over time. At 3 h, 278 DEGs were observed with respect to 0 h (Fig. 3A; Table S2); of these genes, only 67 were different with respect to those observed later, at 9 h, when 823 DEGs with respect to 0 h were observed (Fig. 3A; Table S3); of this set of genes, 612 were different with respect to those observed at 3 h (Fig. 3A). These experiments were performed in MM with none of the carbon source supplemented; for these reasons, many of the DEGs observed could be related to a condition of nutrient limitation.

In the experimental condition supplemented with 0.2 mM MP at 0 h, the transcriptional profile of the experimental condition with MP showed eight DEGs (Fig. 3B; Table S4); with respect to the condition without MP, these genes were also observed at 3 and 9 h. At the 3 h sampling time, a set of 376 DEGs were observed with respect to 0 h (Fig. 3B; Table S5); of these genes, 66 were different in comparison with the DEGs at 9 h. Lastly, at 9 h, 756 DEGs were observed with respect to 0 h (Fig. 3B; Table S6); of this set of genes, 446 were different with respect to those observed at 3 h (Fig. 3B), and several of the observed DEGs may be implicated in the response to MP and PNP exposure.

### Metabolic pathways of *B. zhejiangensis* CEIB S4-3

Based on the KEGG database, 997 DEGs were involved in 104 pathways. In the first sampling time (0 h) in the culture medium with MP, none of the DEGs observed were located in any metabolic pathway. However, in the following sampling time, our results showed that exposure to MP regulates the expression of several genes involved in different metabolic pathways. For example, in the antibiotics biosynthesis, the enzyme dTDP-4-dehydrorhamnose reductase interestingly increased its level of expression in the presence of MP when PNP reached its maximum concentration (3 h) (Table 1). Enzymes from the tricarboxylic acid cycle (TCA), such as succinate dehydrogenase/fumarate reductase, succinyl-CoA, acetate CoA-transferase, citrate synthase, aconitate hydratase, and succinyl-CoA synthetase, were also identified. The MP exposure of the strain also increased the expression of enzymes from other important metabolic pathways, including oxidative phosphorylation, carbon metabolism, butanoate metabolism, ribosome, valine, leucine and isoleucine degradation, lysine degradation, propanoate metabolism, 2-oxocarboxylic acid metabolism, glycolysis/gluconeogenesis, chloroalkane and chloroalkene degradation. On the other hand, in the MM without MP cultures, the metabolic pathways regulated were quorum sensing (QS), ABC transporters, two-component systems, fatty acid metabolism, and amino acid biosynthesis. However, some metabolic pathways were expressed in both conditions with no significant differences in their percentages (Table 1; Tables S7–S10).

**Table 1:** Distribution of the DEGs of *Burkholderia zhejiangensis* CEIB S4-3 at 3 and 9 h in KEGG.

Metabolic pathway	3 h		9 h	
	MP*	WMP†	MP*	WMP†
Biosynthesis of antibiotics	52	9	111	51
Biosynthesis of secondary metabolites	48	20	113	57
Quorum sensing	37	49	139	99
Oxidative phosphorylation	24	5	38	15
Biosynthesis of amino acids	22	10	47	19
Two-component system	21	12	41	51
Biosynthesis of unsaturated fatty acids	19		19	19
Biotin metabolism	19		19	19
Fatty acid biosynthesis	19		19	19
Fatty acid metabolism	19		39	19
Phenylalanine metabolism	19	14	37	
Carbon metabolism	17	6	65	25
Chloroalkane and chloroalkene degradation	17		20	1
ABC transporters	16	37	118	106
Glycine, serine, and threonine metabolism	16	2	25	18
Ribosome	15	2	38	
2-Oxocarboxylic acid metabolism	14		24	
Degradation of aromatic compounds	14	6	17	5
Arginine and proline metabolism	13		13	
C5 Branched dibasic acid metabolism	13		15	
Glycolysis/gluconeogenesis	13		22	10
Purine metabolism	13	4	18	12
Valine, leucine, and isoleucine biosynthesis	13		16	4
Valine, leucine, and isoleucine degradation	13		33	5
Butanoate metabolism	12		43	
Pyruvate metabolism	12		29	5
Ascorbate and aldarate metabolism	11		11	3
Chlorocyclohexane and chlorobenzene degradation	10		10	4
Flagellar assembly	10	2	10	1
Tryptophan metabolism	10	4	31	5
Beta Alanine metabolism	9	3	19	
Fatty acid degradation	9	5	32	1
Glycerolipid metabolism	9		14	
Histidine metabolism	9		9	4
Limonene and pinene degradation	9		15	
Lysine degradation	9		31	
Pyrimidine metabolism	9	4	15	2
Citrate cycle (TCA cycle)	7		22	5
Glyoxylate and dicarboxylate metabolism	7	7	35	12
Methane metabolism	7		16	6
Benzoate degradation	6		28	5
Cysteine and methionine metabolism	6	4	7	7
Phosphonate and phosphinate metabolism	6		6	
Synthesis and degradation of ketone bodies	6		15	
Galactose metabolism	5		4	4
Nitrogen metabolism	5	11	9	12
One carbon pool by folate	5		7	
Dioxin degradation	4		4	
Fluorobenzoate degradation	4		4	5
Folate biosynthesis	4		4	4
Naphthalene degradation	4		7	1
Polycyclic aromatic hydrocarbon degradation	4		4	3
Toluene degradation	4	1	4	5
Beta-Lactam resistance	3		10	
Cyanoamino acid metabolism	3		3	
Novobiocin biosynthesis	3		3	
Phenylalanine, tyrosine, and tryptophan biosynthesis	3	3	15	4
Propanoate metabolism	3		27	6
Taurine and hypotaurine metabolism	3		4	
Tyrosine metabolism	3	1	13	1
Alanine, aspartate, and glutamate metabolism	2	5	12	3
Bacterial chemotaxis	2		17	9
Carbon fixation in photosynthetic organisms	2		5	2
Pentose phosphate pathway	2		2	4
Polyketide sugar unit biosynthesis	2		2	
RNA degradation	2	2	4	6
Streptomycin biosynthesis	2		2	
Ubiquinone and other terpenoid quinone biosynthesis	2		5	2
Aminobenzoate degradation	1	5	7	6
Arginine biosynthesis	1	3	3	3
Fructose and mannose metabolism	1	1	2	4
Lysine biosynthesis	1		1	
Porphyrin and chlorophyll metabolism	1		5	4
Riboflavin metabolism	1		3	
RNA polymerase	1		3	
Pantothenate and CoA biosynthesis		11	4	8
Nicotinate and nicotinamide metabolism		5	8	2
Starch and sucrose metabolism		5		8
Sulfur metabolism		5	7	9
Non homologous end joining		3		3
Amino sugar and nucleotide sugar metabolism		2		2
Glycerophospholipid metabolism		1	3	2
Styrene degradation		1	3	1
Pentose and glucuronate interconversions		1	5	1
Xylene degradation		1		1
Base excision repair				1
Nucleotide excision repair				1
D-Glutamine and D-glutamate metabolism			1	
Aminoacyl tRNA biosynthesis			1	
alpha-Linolenic acid metabolism			1	
Thiamine metabolism			2	
Monobactam biosynthesis			2	
DNA replication			3	2
Inositol phosphate metabolism			3	
Bacterial secretion system			3	4
Selenocompound metabolism			3	4
Mismatch repair			3	1
Protein export			4	1
Homologous recombination			4	
Caprolactam degradation			7	
Geraniol degradation			7	
Cationic antimicrobial peptide (CAMP) resistance			8	
Terpenoid backbone biosynthesis			9	1
Glutathione metabolism			14	13

**Note:**

The numbers represent the genes of the metabolic pathway in each condition.

* MP, methyl parathion to 0.2 mM.

† WMP, without methyl parathion.

### Distribution of COGs by condition

We analyzed DEGs in order to identify whether they participate in different functions, or if they were distributed in a small number of functional categories. DEGs were classified according to the functional COGS of proteins (Galperin et al., 2014).

**Figure 4:**
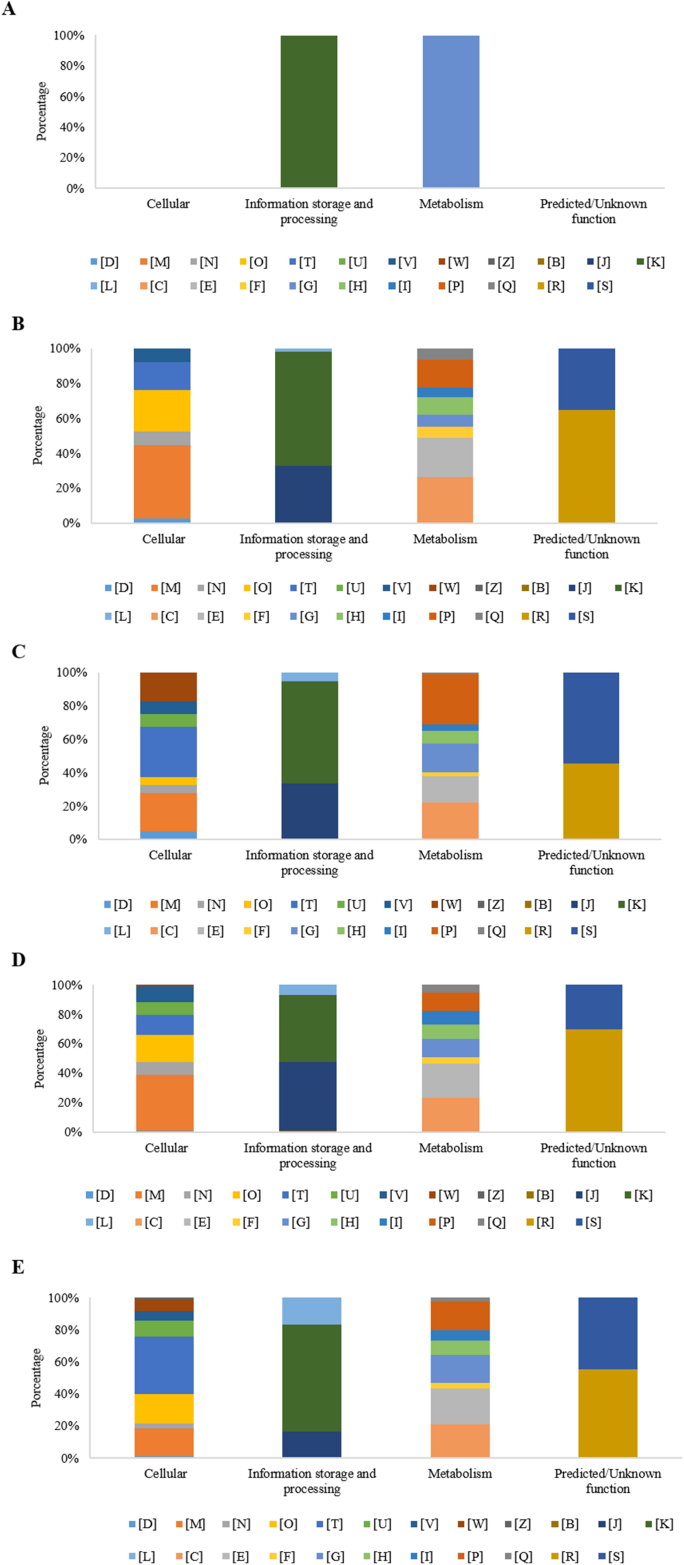
Percentage of differential expressed genes (DEGs) observed by condition and time, organized according to their associated COGs functional categories. (A) Time 0 h with MP; (B) Time 3 h with MP; (C) Time 3 h without MP; (D) Time 9 h with MP; and (E) Time 9 h without MP. [B] Structure and dynamics of chromatin; [C] Production and energy conversion; [D] Control of the cell cycle, cell division, chromosomal partitioning; [E] Amino acid transport and metabolism; [F] Nucleotide transport and metabolism; [G] Carbohydrate transport and metabolism; [H] Coenzyme transport and metabolism; [I] Lipid transport and metabolism; [J] Translation, ribosomal structure, and biogenesis; [K] Transcription; [L] Replication, recombination, and repair; [M] Cell wall/membrane/envelope biogenesis; [N] Cell motility; [O] Post-translational modification, protein turnover, chaperones; [P] Inorganic ions transport and metabolism; [Q] Secondary metabolites biosynthesis, transport, and catabolism; [R] General function prediction only; [S] Function Unknown; [T] Signal transduction mechanisms; [U] Intracellular trafficking, secretion, and vesicular transport; [V] Defense mechanisms; [W] Extracellular structures; [X] Mobiloma: prophages, transposons; [Z] Cytoskeleton.

At 0 h, in the experimental condition with MP, eight DEGs were observed. Five of those genes did not group into a functional category (62%), while the three remaining genes (38%) presented two COGs grouped into the functional [K] category (transcription), and one into the [G] category (carbohydrate transport and metabolism) (Fig. 4A).

After 3 h, in the experimental condition with MP, 376 DEGs were observed, of which, 271 genes were grouped into 20 COGs (72.3%) and 104 genes (27.7%) were not grouped into any category. The overrepresented categories were energy production and conversion [C] with 38 genes (14.0%), 36 genes in the transcription category [K] (13.3%), 32 genes in the amino acid transport and metabolism category [E] (11.8%), 23 genes for the inorganic ion transport and metabolism category [P] (8.5%), 22 genes with only a general function prediction [R] (8.1%), 18 genes for the translation, ribosomal structure and biogenesis category [J] (6.6%), 16 genes for the cell wall/membrane/envelope biogenesis category [M] (5.9%), 14 genes for the coenzyme transport and metabolism category [H] (5.2%), and 12 genes with no function assigned [S], representing 4.4% (Fig. 4B). On the other hand, in the experimental condition MM without MP, a set of 278 DEGs was found, of which 158 genes (56.8%) were grouped into 22 COGs, and the remaining 120 genes (43.2%) were not grouped into any category. Regarding those grouped into COGs, the five principal functional categories that include the highest number of DEGs were inorganic ion transport and metabolism [P], (23 genes, 14.6%); 17 genes in the energy production and conversion category [C], (10.8%); 13 genes in carbohydrate transport and metabolism [G], (8.2%); 12 genes in the signal transduction mechanisms category [T] (7.6%); and 12 genes with no function assigned [S], representing 7.6% (Fig. 4C).

As is shown in Fig. 4D, at 9 h, the experimental condition MM with MP showed a set of 756 DEGs. Of these genes, 556 (73.5%) were grouped in 23 COGs, while the remaining 200 genes (26.5%) were not grouped into any category. The five main overrepresented categories (70 genes, 12.6%) were the following categories: [E], amino acid transport and metabolism, 69 genes (12.4%); [C], energy production and conversion, 49 genes (8.8%); [J], translation, ribosomal structure, and biogenesis, 48 genes (8.6%); [K], transcription; and 39 genes (7.0%) for the [M] category, cell wall/membrane/envelope biogenesis.

At the same sampling time, in the condition without MP, a set of 823 DEGs was found, where a subset of 427 genes (51.95%) was grouped in 23 COGs and 396 genes (48.1%) were not classified into any category. In the subset of 427 DEGs that showed COGs, a higher number were grouped in the following functional categories: 46 genes (10.8%) showed only a general function prediction, category [R]; 40 genes for the [K] category, transcription (9.4%); 39 genes for the [E] category, amino acid transport and metabolism (9.1%); 38 genes for the [T] category, signal transduction mechanisms (8.9%); and 37 genes with no function assigned [S] representing 8.7% (Fig. 4E).

### Analysis of the relative expression of *mpd* gene

**Figure 5:**
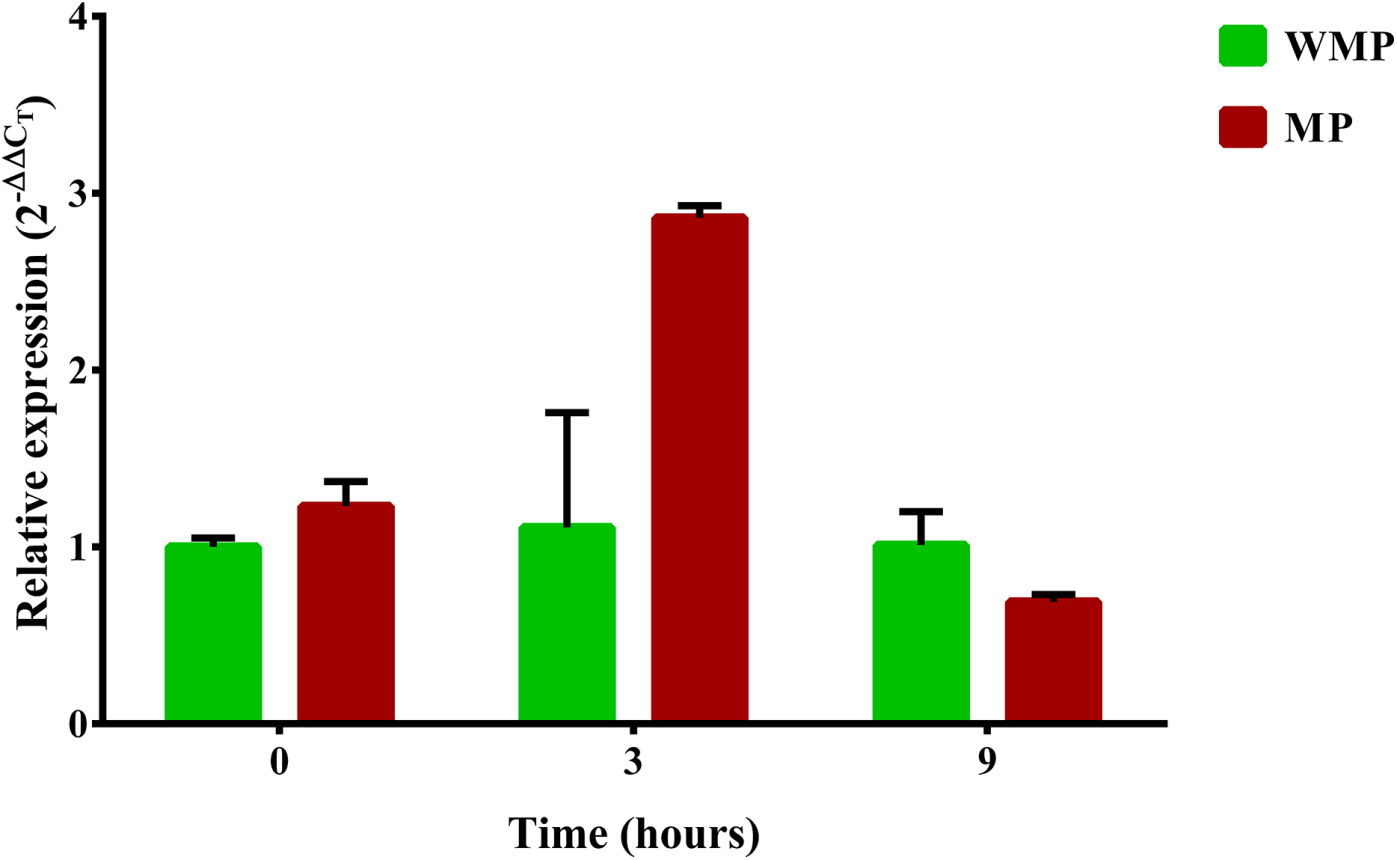
Differential expression of the *mpd* gene observed by condition and time. Cells were collected at three sampling times to extract total RNA. The expression level of the *mpd* gene in MP and MM presence were measured by qRT-PCR and calculated through the 2^−ΔΔ*C*^_*T*_ method. Bars in green represent the condition without MP and bars in red the condition with pesticide. The values are averages of results from three independent experiments, error bars represent the standard deviations. According to the results of Unpaired *t-*test analysis, no significant differences in the relative expression of the gene *mpd* were found between both experimental conditions at sampling time 0 h (*p* = 0.0552), in the time 3 h, the relative expression of the gene *mpd* increased significatively in presence of the pesticide (*p* = 0.0098) while at sampling time 9 h the relative expression of *mpd* gene decreased significantly (0.0462) in the same condition. WMP, experimental condition in absence of MP; MP, experimental condition in the presence of MP.

One relevant aspect of this research was to evaluate the gene expression involved in the complete degradation of MP. For this reason, a specific search of these genes and their expression profiles was carried out. The first step in the process of biodegradation of MP is its hydrolysis into dimethylthiophosphoric acid and PNP. In different bacterial strains, this enzymatic activity is the responsibility of the *mpd* gene, which codes for MPH (Li, He & Li, 2007; Yang et al., 2008). In a previous report, the *B. zhejiangensis* CEIB S4-3 *mpd* gene was identified and later recombinantly expressed in *E. coli* BL21 (DE3) pLysS; the crude extract of the bacterial expression was experimentally evaluated for MP hydrolysis when the release of PNP was observed, which suggested that MPH was present (Popoca-Ursino et al., 2017). In this work, the relative expression (2^−ΔΔ*C*^_*T*_) of the *mpd* gene was evaluated by qRT-PCR. The relative gene expression in the experimental condition without MP showed a value of 1 in the three sampling times, while in the presence of MP, the relative gene expression was 1.23, 2.86, and 0.69 at 0, 3, and 9 h, respectively (Fig. 5).

### Transcriptional analysis of clusters *pnpABA′E1E2FDC* and *pnpE1E2FDC* involved in PNP degradation

**Figure 6:**
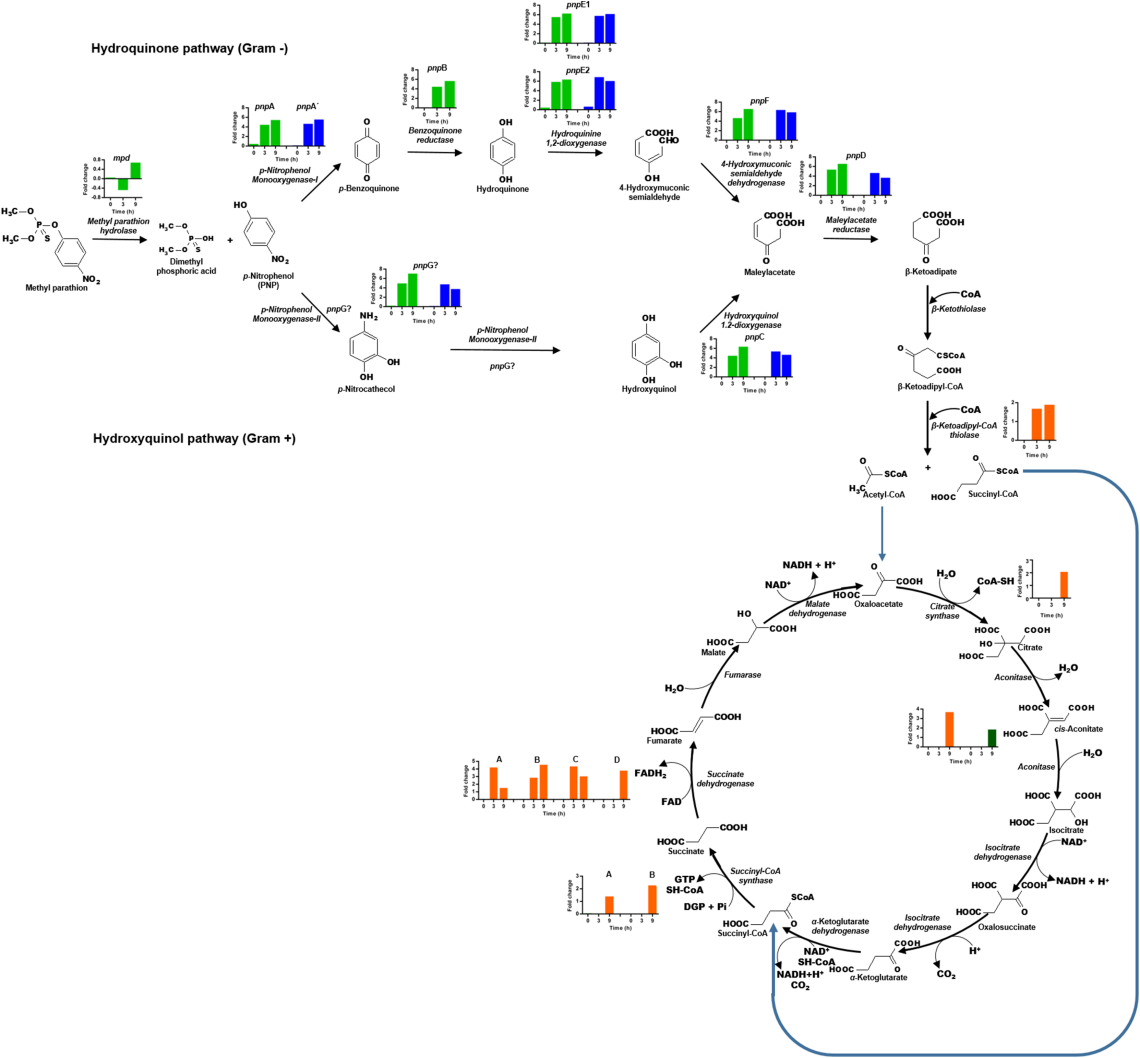
Differential expression of the clusters of genes involved in PNP degradation by *Burkholderia zhejiangensis* CEIB S4-3. Green bar charts represent the fold change values of the genes from cluster *pnpABA′E1E2FDC* and in blue the fold change values of the genes from cluster *pnpE1E2FDC.* The fold change values of enzymes related to the use of PNP biodegradation metabolites in TCA pathway are shown in orange and dark-green colors.

The transcriptional profile of the eight genes that form the cluster *pnpABA′E1E2FDC*, located in Contig 33 of the strain, was analyzed (Fig. 6). In general, the genes of this cluster present higher expression levels as time increases. The *pnpA* gene encodes the enzyme PNP monooxygenase I, which was the first reported enzyme in the PNP degradation pathway; it is responsible for the oxidation of PNP to produce benzoquinone. The *pnpA* gene was differentially expressed since 0 h, with a fold change of 0.5; then, the expression profile increased in time, and at 3 and 9 h, it showed a fold change value of 4.5 and 5.5, respectively. On the other hand, the *pnpB* gene (benzoquinone reductase) presented a fold change of 0.06, 4.6, and 5.8 at 0, 3, and 9 h, respectively. For the *pnpA′*, *pnpE1*, *pnpE2* (hydroquinone 1,2-dioxygenase) genes, the same increasing expression pattern was found, and fold change values were obtained as follows: 0 h = −0.1, 0.2, 0.5; 3 h = 4.7, 5.6, 5.9 and 9 h = 5.6, 6.3, 6.4, respectively. Meanwhile, for the genes *pnpF* (4-hydroxyuconic semialdehyde dehydrogenase), *pnpD* (maleiloacetate reductase), and *pnpC* (hydroxyquinol 1,2-dioxygenase), the fold change values were as follows: 0 h = −0.06, −0.101, 0.057; 3 h = 4.7, 5.4, 4.5; and finally, for 9 h, rising values of 6.6, 6.6, and 6.4, respectively. In addition to the PNP degrading cluster, the *pnpG* gene has a possible PNP monooxygenase-II activity (Popoca-Ursino et al., 2017), in this work the expression levels of this gene increased over the time, showing values of fold change of 0.25, 5.0, and 7.1 for the times 0, 3, and 9 h respectively.

In addition, for the five genes of cluster *pnpE1E2FDC*, which is located in Contig 4 of *B. zhejiangensis* CEIB S4-3, it was observed that the *pnpE1* gene was differentially expressed at the three sampling times, obtaining fold change values of 0.2, 5.8, and 6.2 for the times of 0, 3, and 9 h, respectively. However, for the genes *pnpE2*, *pnpF*, *pnpD*, *pnpC*, and *pnpG*, the expression increased from fold change values of 0.7, 0.1, 0.04, 0.1, 0.2 at time 0 h to 6.9, 6.4, 4.7, 5.4 at 3 h; while at 9 h, the gene expression decreased to fold change values of 6.1, 5.9, 3.7, 4.7, and 3.8, respectively (Fig. 6). The genes of this cluster showed greater expression at the 3 h time point, precisely when the PNP concentration in the culture media reached its maximum value.

The relative changes in the gene transcriptional levels implicated in the PNP degrading clusters *pnpABA′E1E2FDC* and *pnpE1E2FDC* were evaluated by qRT-PCR. The relative expression of these genes was comparable with that observed in the transcriptomic analysis. In the experimental condition without MP, the genes of the PNP degrading clusters show a relative expression of 1.0 in all sampling times. While, in the condition with MP the qRT-PCR results show an increase in the expression of all the genes. One of the genes with the greatest expression was the gene *pnpA′*, with a value of 1.57 at 0 h, which then increased with time, with 625.93 and 201.61 at 3 and 9 h, respectively (Table 2).

**Table 2:** Expression profiles of the two PNP degradation gene clusters in *Burkholderia zhejiangensis* CEIB S4-3.

Gene name	Function	0 h	3 h	9 h
		qRT-PCR (MP)		
*pnpA*	*p*-nitrophenol monooxygenase I	2.02 ± 0.53	81.86 ± 12.40	63.96 ± 7.11
*pnpA′*	*p*-nitrophenol monooxygenase I	1.57 ± 1.37	625.93 ± 187.32	201.61 ± 37.12
*pnpB*	Benzoquinone reductase	1.35 ± 0.24	236.76 ± 8.60	112.14 ± 7.96
*pnpE2*	Hydroquinone 1,2-dioxygenase	3.71 ± 0.60	243.34 ± 4.44	98.92 ± 5.52
*pnpE1*	Hydroquinone 1,2-dioxygenase	2.55 ± 0.62	159.95 ± 44.03	97.60 ± 6.41
*pnpF*	4-Hydroxymuconic semialdehyde dehydrogenase	1.25 ± 0.31	99.11 ± 4.36	69.96 ± 4.33
*pnpD*	Maleylacetate reductase	1.07 ± 0.42	99.58 ± 19.95	108.52 ± 27.19
*pnpG*	*p*-nitrophenol monooxygenase II	1.15 ± 0.38	90.74 ± 2.28	62.78 ± 4.21
*pnpC*	Hydroxyquinol 1,2-dioxygenase	1.01 ± 0.15	94.59 ± 2.46	90.00 ± 5.64

**Note:**

qRT-PCR, quantitative reverse transcription polymerase chain reaction; MP, methyl parathion.

The hydrolysis of MP releases two metabolites, namely, dimethylthiophosphoric acid and PNP. *B. zhejiangensis* CEIB S4-3 can probably metabolize MP and its degradation metabolite dimethylthiophosphoric acid as a phosphorous source and PNP as a carbon and energy source (Serdar et al., 1982; Chakrabarty, 2017). In this work, the enzyme β-ketoadipyl-CoA thiolase, also known as 3-oxoadipyl thiolase, was identified; this enzyme increased its transcriptional levels in the presence of MP and is involved in the biodegradation of benzoate through hydroxylation; it also synthetizes the conversion of β-ketoadipyl-CoA to acetyl-CoA and succinyl-CoA. These metabolites are intermediates in the TCA cycle, as shown in Fig. 6.

Besides, different enzymes from the TCA cycle increased their expression levels when the pesticide was present in the media. The *gltA* gene corresponding to the enzyme citrate synthase increased its transcriptional level, this enzyme catalyzed the first reaction of the metabolic cycle, that is, the condensation of acetyl-CoA and oxaloacetate to citrate; two genes of the enzyme aconitase are involved in the bioconversion of citrate to isocitrate via *cis*-aconitate, and these also increased their transcriptional levels. Another enzyme of the TCA cycle that increased its transcriptional levels was succinyl-CoA synthetase; this enzyme catalyses the bioconversion of succinyl-CoA to succinate. Finally, the genes that encode four subunits of succinic dehydrogenase, an enzyme that catalyses the formation of fumarate using the succinate generated in the prior step of the pathway as a substrate, were found.

Since MP was used as the sole carbon source, after its hydrolysis, the generated PNP could be channeled to the TCA cycle for energy generation and carbon distribution in different metabolic pathways. The overexpression of these enzymes in the presence of MP could play an important role in conducting the acetyl-CoA and succinyl-CoA generated by the degradation of PNP to the TCA cycle for use as a carbon and energy source.

## Discussion

In this work, we report a transcriptomic study over *B. zhejiangensis* CEIB S4-3, a strain isolated from agricultural soils in Mexico, which is capable of immediately hydrolyzing MP and completely degrading PNP in 12 h without requiring an additional carbon source. In a previous study, Popoca-Ursino et al. (2017), using this bacterial strain, reported the complete degradation of PNP in a time of 15 h using an OD_600 nm_ of 0.15 culture. The discrepancy between the PNP biodegradation times, can be explained by the higher bacterial population evaluated in this report, in which an OD_600 nm_ of 0.5 culture was used.

While several studies report on bacteria isolated from contaminated soils that are capable of hydrolyzing MP and degrading PNP separately, reports on bacteria that are capable of degrading both compounds are scarce (Pakala et al., 2007). Bara et al. (2017) report that *B. cepacia* rapidly degrades MP and PNP and can utilize them as sole carbon sources; however, in the same report, other bacteria such as *Pseudomonas* sp., are mentioned to hydrolyze the pesticide to PNP, but require glucose or another carbon source for growth. *Flavobacterium* sp. metabolizes PNP releasing nitrite to supply nitrogen for bacterial metabolism, and *Bacillus* sp., which can hydrolyze MP in the presence of different concentrations of yeast extract. Mishra, Khan & Pandey (2017) observed that the utilization of MP by *Achromobacter xylosoxidans* reached a maximum when glucose was used as a carbon source and NH_4_Cl was used as a nitrogen source.

According to genomic data, *B. zhejiangensis* CEIB S4-3 showed a set of genes, that is, implicated in PNP degradation through both reported metabolic pathways (Hernández-Mendoza et al., 2014). The presence of these genes in *B. zhejiangensis* CEIB S4-3 raises the hypothesis of that this strain carries out PNP degradation using both metabolic mechanisms. Has been reported that other species from the proteobacteria group as *Burkholderia* sp. strain SJ98 (Vikram et al., 2012) and *Pseudomonas* sp. 1–7 (Zhang et al., 2012) can carry out PNP biodegradation by employing both metabolic pathways.

As was reported by Chauhan et al. (2010) and Vikram et al. (2012), *Burkholderia* sp. SJ98, a Gram-negative bacterium, is capable of carrying out PNP degradation through the hydroquinone (HQ) by open reading frames (ORFs) *pnpE1* and *pnpE2* and benzenetriol (BT) by ORFs *pnpC* and *pnpD* and is able to use it as the sole source of carbon, nitrogen, and energy (Min, Wang & Hu, 2017). Another study reported by Zhang et al. (2012) states that *Pseudomonas* sp. 1–7 is a Gram-negative bacterium that degrades PNP by two reported pathways, the HQ by the gene cluster *pdcABDEF* and genes *pdcCG* in the BT pathway. *Pseudomonas* sp. strain WBC-3 is capable of utilizing MP or PNP as the sole source of carbon, nitrogen, and energy using a plasmid designated as pZWL0, which is responsible for MP and PNP degradation (Liu et al., 2005). On the other hand, Zhang et al. (2009) reported that the *pnpABCDEF* gene cluster of strain WBC-3 is involved in PNP degradation, and *Pseudomonas putida* strain DLL-E4 also revealed that the *pnpRC1C2DECX1X2* and *pnpA* region of the *pnp* gene cluster contained all of the essential genes involved in HQ and BT degradation (Shen et al., 2010; Chen et al., 2016).

While degradation of MP takes place, other processes occur in the cell, especially transcriptional regulation. In presence of MP, transcriptomic data reveals the induction of genes involved in several cellular processes, including genes that codify efflux pumps, porins, permeases, transcriptional regulators and transporters involved in multidrug resistance. Chakka et al. (2015) reported that transcriptional regulation is not limited only to regulation of the genes involved in PNP degradation but to a whole set of metabolic and transcriptional processes. In this study, as a result of the presence of MP, different transcriptional regulator showed an induction, the number of these kinds of genes, that showed an overexpression increases as the culture time increases (two genes at 0 h, 29 genes at 3 h, and 41 genes at 9 h).

Chen et al. (2016) mention multiple components, including transcriptional regulators and other unknown factors that regulate PNP degradation in *Pseudomonas putida* DLL-E4, as the transcriptional regulator type LysR (LTTR) activates the expression of genes in response to the specific inducer PNP. Also, Wang et al. (2017) mention that a LTTR, PnpR, has previously been shown to activate the transcription of operons *pnpABCDEFG* for PNP degradation in *Pseudomonas* sp. strain WBC-3. A regulator of this type is present in *B. zhejiangensis* CEIB S4-3 and presents overexpression in the condition with MP. LTTRs are found in diverse bacterial genera. They are associated with the regulation of degradation pathways of aromatic compounds and control the gene expression of the catechol (CatR) and Chlorocatechol (ClcR and CbnR) *ortho*-cleavage pathways (Díaz & Prieto, 2000; Tropel & Van Der Meer, 2004). Similarly, multiple antibiotic resistance regulator regulates the activity of genes involved in antibiotic resistance, stress responses, virulence, or catabolism of aromatic compounds (Egland & Harwood, 1999; Kim et al., 2006; Perera & Grove, 2010).

Another transcriptional regulator that showed differential expression was IclR, the members of this family are repressors of the isocitrate lyase gene (Romero-Rodríguez, Robledo-Casados & Sánchez, 2015). They play diverse functions, including carbon metabolism in Enterobacteriaceae, degradation of aromatic compounds by soil bacteria, and tolerance to solvents in *Pseudomonas* genus (Krell, Molina-Henares & Ramos, 2006). In this work the overexpression of other families of transcriptional regulators, as GntR, AraC, and MerR, was observed in presence of the pesticide. GntR family transcriptional regulators are widely distributed throughout bacteria. They regulate many diverse biological processes including fundamental cellular processes such as motility, development, antibiotic production, antibiotic resistance, plasmid transference, virulence, and the degradation of aromatic compounds (Díaz & Prieto, 2000; Tropel & Van Der Meer, 2004; Hoskisson & Rigali, 2009). The best characterized AraC transcriptional regulators family member in catabolism of aromatic compounds is the XylS protein. This protein positively regulates the *Pm* promoter, which derives in the expression of the TOL *meta*-cleavage pathway for aromatic compounds (Díaz & Prieto, 2000). Finally, the MerR family is a group of transcriptional activators, respond to environmental stimuli, such as oxidative stress, heavy metals, or antibiotic presence (Brown et al., 2003). In a future study the functional verification of these transcriptional regulator and their role in the PNP biodegradation could be evaluated.

Differentially expressed gene analysis results indicate that *B. zhejiangensis* CEIB S4-3 induces genes that encode hypothetical proteins and proteins of unknown function in the culture medium with MP, representing 50%, 20%, and 15% for the initial time (0 h), 3 and 9 h, respectively. In the condition without MP, the induction of these genes was observed, representing 33% and 36% at 3 and 9 h, respectively. Chen et al. (2016) carried out a transcriptomic analysis of *Pseudomonas putida* DLL-E4 in the presence of PNP. They reported a greater number of genes that corresponded to hypothetical proteins and proteins with unknown function as was observed in the present work.

According to the COG analysis, the strain *B. zhejiangensis* CEIB S4-3, in the condition with MP, at 3 and 9 h, showed an overexpression of genes that codify proteins included mainly in the following functional categories: energy production and conversion [C] (14.0% and 12.4%, respectively), transport and amino acid metabolism [E] (11.8% and 12.6%, respectively) and post-translational modification, protein replacement, and chaperones [O] (3.3% and 3.4%, respectively). Similar findings were reported by Liao et al. (2018), where the capability of *Citrobacter* sp. to degrade 2,4,6-trinitrotoluene (TNT) was evaluated using a transcriptomic approach. They observed after 12 h, in presence of TNT, that the set of upregulated proteins were grouped, from higher to lower percentage, in three main functional categories [C], [E], and [O], as was observed in this study.

In another study, Cheng et al. (2018) reported the gene expression changes during the degradation of chlorimuron-ethyl by *Rhodococcus erythropolis* D310-1. The RNA-Seq results revealed that 500 genes were upregulated during chlorimuron-ethyl degradation, and COGs annotation showed that the total 2,978 unigenes were classified into 24 COG categories. Among the COG categories, the cluster for [R] general function prediction represented the largest group (27.54%), followed by [E] amino acid transport and metabolism (18.49%), [Q] secondary metabolites biosynthesis, transport and catabolism (17.09%), [I] lipid transport and metabolism (16.49%), [K] transcription (15.92%), [P] inorganic ion transport and metabolism (14.92%), [C] energy production and conversion (12.32%), [G] carbohydrate transport and metabolism (11.42%), [S] function unknown (10.14%). The categories [B] chromatin structure and dynamics and [A] RNA processing and modification are both associated with 0.04% of the unigenes, represented the smallest groups. *B. zhejiangensis* CEIB S4-3 cultured with MP showed an overrepresentation of most of the COGs, such as [K], [C], [E], [G], [P], [R], [Q], [I], [B], and [A], which coincides with this report. The overexpression of these functional categories suggests that the pesticide presence generates transcriptional changes that allow the cell to obtain energetic resources through essential pathways, as well as carbon distribution in metabolic pathways and the ability to contend with oxidative stress.

Under the experimental conditions of this work, *B. zhejiangensis* CEIB S4-3 bacterial cells were exposed to different stress conditions, such as the limitation of carbon source or the presence of a non-conventional carbon source such as MP. In response to these conditions, the cells overexpress different processes and metabolic pathways. One of them is QS, which is a communication mechanism between bacteria (Sibanda et al., 2018), that controls specific defence processes, such as biofilm formation, virulence factor expression, secondary metabolite production, and mechanisms to contend with the stress (Barreto, 2013). In different microorganisms, it has been reported that the limitation of a carbon source activates QS mechanisms, which is a situation that promotes the microbial population in the culture to start a physiological state characteristic of the stationary phase of growth (Lazazzera, 2000). Yong & Zhong (2010) reported that using newly constructed biosensor acylated homoserine lactone, QS signals have been detected and identified during aromatic biodegradation.

On the other hand, in this work, especially at the time 9 h, the ABC transporters showed high level expression in both conditions, with and without MP. ABC transporters, also known as ATP-dependent transporters, which are a group of proteins, bound the bacterial membrane and needed ATP hydrolysis to carry out the transport function. These proteins participate in the transport of several metabolites and ions, including sugars, amino acids, metallic ions, peptides, and proteins, as well the export of toxic molecules to the extracellular media (Davidson et al., 2008; Couoh-Uicab, Islas-Flores & Canto-Canché, 2010). These kinds of cellular transporters have established roles in the resistance to toxins and xenobiotics (De Waard et al., 2006). Nevertheless, despite the fact that the expression of this kind of transporters is slightly higher in the presence of the pesticide, the role of PNP as inductor of these genes is not conclusive according to the observed data.

Our results showed that the overexpression of the two-component system in both experimental conditions. Studies have reported that the two-component system regulates the bacterial physiology according to environmental conditions, such as nitrogen limitation and the presence of xenobiotics compounds. Tiwari et al. (2018) reported the importance of this signaling system as a strategy for the tolerance and biodegradation of MP in the cyanobacterium *Fischerella* sp. In this study, we report the positive regulation of hypothetical proteins in the presence of MP. In this report, we mention that two components of the regulatory system transmit the phosphorylation signal to a hypothetical protein, in a direct or indirect way, and this hypothetical protein serves as a possible transcriptional factor. According to this, it is possible that this protein could induce the expression of different stress response proteins, including a hypothetical hydrolase with the capability of joining carbohydrates and MP. This suggested that this protein plays a crucial role in the biodegradation of the pesticide. However, because the overexpression of the two-component system in *B. zhejiangensis* CEIB S4-3 was observed in both experimental conditions, it is feasible that the observed overexpression of this signaling system could be related with the nutrients limitation, not by the presence of MP or its hydrolysis metabolites.

The activation of carbon metabolism could be related to the use of alternative carbon sources from cellular reserves. In this work, the overexpression of genes that encode enzymes included in butanoate metabolism, also known as butyrate, was observed. In the *Burkholderia* genus, polyhydroxybutyrate (PHB) is used as a reserve metabolite. PHB is produced by different microorganisms as a carbon assimilation product, generally through glucose or starch consumption, and it is employed by microorganisms as a form of storage of energy to be metabolized when other conventional energy sources are not available in the media. Our results confirmed this phenomenon when MP was the only available carbon and energy source supplemented for bacterial metabolism. Furthermore, an overexpression of fatty acid catabolism was observed, and the use of fatty acid reserves is important for generating energy and carbon skeletons for the synthesis of other metabolites in the context of nutrient limitations. In this work, overexpression of the oxidative phosphorylation pathway, which correlates with the increase in the nutrient oxidation process to produce ATP, was also observed. We can conclude that *B. zhejiangensis* CEIB S4-3 utilizes PNP as energy and carbon source to generate important metabolites for maintenance of the cell population, when pesticides are present.

The use of metabolic reserves not only provides energy for cellular maintenance but also allows to generate carbon skeletons for the synthesis of other important metabolites as amino acids. In this work, an overexpression of the amino acid biosynthesis pathways was observed. Amino acids are necessary to produce proteins that fight with the stress generated by the nutrient limitation and the exposure to MP. Bacteria are required to synthesize amino acids when they are not present in the culture medium, and they are generated through the used glycolytic intermediates, which are metabolites of the TCA cycle or from the pentose phosphate pathway. As with the overexpression in ribosome generation, it is also important to accelerate protein synthesis in stress conditions.

However, the enzyme from dTDP-4-dehydrorhamnose reductase interestingly increased its level of expression in the presence of MP when PNP reached its maximum concentration (3 h). This enzyme belongs to the oxidoreductase family and participates in three metabolic pathways, namely, nucleotide sugar metabolism, streptomycin biosynthesis, and polyketide sugar unit biosynthesis (Srinivasan & Rajamohan, in press). Cao et al. (2018) mention that severe oxidative stress in bacteria may lead to ATP depletion. Therefore, they evaluated the relationship between Mn deficiency and oxidative stress in *Streptococcus pneumoniae* and found that 14 proteins (17.7%) participated in the oxidative stress pathway such as dTDP-4-dehydrorhamnose reductase.

Following PNP degradation by *B. zhejiangensis* CEIB S4-3, in addition to cellular metabolism, different enzymes of the TCA cycle, such as succinate dehydrogenase/fumarate reductase, succinyl-CoA acetate CoA-transferase, citrate synthase, aconitate hydratase, and succinyl-CoA synthetase, also increased their differential expression levels with MP. These results coincide with that reported by Chen et al. (2016), where global changes in the transcriptional profile of *Pseudomonas putida* DLL-E4 caused by exposure to PNP were observed, and enzymes of the TCA cycle were overrepresented when cells were grown on PNP plus glucose. In other report, Chakka et al. (2015) performed a transcriptional analysis of *E. coli* cultivated in presence of PNP, observing a significant decrease in the gene transcription coding for glycolysis and enzymes of the TCA cycle. Therefore, PNP can be channeled to the TCA cycle for energy generation and/or carbon distribution of this metabolite in other metabolic pathways.

## Conclusions

*Burkholderia zhejiangensis CEIB* S4-3 is able to immediately hydrolyze MP and totally degrade PNP in a time of 12 h. According to the transcriptomic analysis, the strain uses the two PNP reported biodegradation metabolic pathways, revealed by the presence of transcripts for enzymes related to both metabolic pathways. The exposure to MP and PNP causes global changes in the gene expression profile, induces the differential expression of genes involved in the pesticide degradation process, carbon metabolism, detoxify, and oxidative stress cell protection. The observed overexpression of genes that codify to transporters, as permeases and porins, suggest that they are indispensable for the transfer of PNP into the cell, in addition to the exchange of molecules that contend with the toxicity of the metabolites generated by the PNP biodegradation.

## Supplemental Information

10.7717/peerj.6822/supp-1 Supplemental Information 1 Primers used for gene validation by qRT-PCR. Primers were designed based on to the nucleotide sequences of the selected genes. The methyl parathion hydrolase (*mpd*) gene, the genes that form *p*-nitrophenol catabolic clusters *pnpABA′E1E2FDC* and *pnpE1E2FDC*, and *pnpG?* gene that codifies for a possible *p*-nitrophenol monooxygenase-II. Recombinase A (*recA*) gene was used as an internal (unregulated) reference for relative quantification.

10.7717/peerj.6822/supp-2 Supplemental Information 2 DEGs at the time 3 h without MP. For the determination of DEGs at the time 3 h without MP, a fold change analysis was carry out, the genes with differential expression profile were those that showed values of ±1.5 (*p*-value < 0.05). Data were used to construct Table 1 and Figs. 3–6.

10.7717/peerj.6822/supp-3 Supplemental Information 3 DEGs at the time 9 h without MP. For the determination of DEGs at the time 9 h without MP, a fold change analysis was carry out, the genes with differential expression profile were those that showed values of ±1.5 (*p*-value < 0.05). Data were used to construct Table 1 and Figs. 3–6.

10.7717/peerj.6822/supp-4 Supplemental Information 4 DEGs at the time 0 h with MP. For the determination of DEGs at the time 0 h with MP, a fold change analysis was carry out, the genes with differential expression profile were those that showed values of ±1.5 (*p*-value < 0.05). Data were used to construct Table 1 and Figs. 3–6.

10.7717/peerj.6822/supp-5 Supplemental Information 5 DEGs at the time 3 h with MP. For the determination of DEGs at the time 3 h with MP, a fold change analysis was carry out, the genes with differential expression profile were those that showed values of ±1.5 (*p*-value < 0.05). Data were used to construct Table 1 and Figs. 3–6.

10.7717/peerj.6822/supp-6 Supplemental Information 6 DEGs at the time 9 h with MP. For the determination of DEGs at the time 9 h with MP, a fold change analysis was carry out, the genes with differential expression profile were those that showed values of ±1.5 (*p*-value < 0.05). Data were used to construct Table 1 and Figs. 3–6.

10.7717/peerj.6822/supp-7 Supplemental Information 7 Metabolic pathways corresponding to the time 3 h with MP. The DGEs were used to locate the knockout (KO) genes. Later the KO numbers were analyzed in the Kyoto Encyclopedia of Genes and Genomes (KEGG) Pathway Color database to identify the metabolic pathways represented at the time 3 h in the experimental condition supplemented with the MP. Data were used to construct Fig. 6 and for Discussion section.

10.7717/peerj.6822/supp-8 Supplemental Information 8 Metabolic pathways corresponding to the time 9 h with MP. The DGEs were used to locate the knockout (KO) genes. Later the KO numbers were analyzed in the Kyoto Encyclopedia of Genes and Genomes (KEGG) Pathway Color database to identify the metabolic pathways represented at the time 9 h in the experimental condition supplemented with the MP. Data were used to construct Fig. 6 and for Discussion section.

10.7717/peerj.6822/supp-9 Supplemental Information 9 Metabolic pathways corresponding to the time 3 h without MP. The DGEs were used to locate the knockout (KO) genes. Later the KO numbers were analyzed in the Kyoto Encyclopedia of Genes and Genomes (KEGG) Pathway Color database to identify the metabolic pathways represented at the time 3 h in the experimental condition without MP. Data were used to construct Fig. 6 and for Discussion section.

10.7717/peerj.6822/supp-10 Supplemental Information 10 Metabolic pathways corresponding to the time 9 h without MP. The DGEs were used to locate the knockout (KO) genes. Later the KO numbers were analyzed in the Kyoto Encyclopedia of Genes and Genomes (KEGG) Pathway Color database to identify the metabolic pathways represented at the time 9 h in the experimental condition without MP. Data were used to construct Fig. 6 and for Discussion section.

10.7717/peerj.6822/supp-11 Supplemental Information 11 Dataset S1. Relative expression of *mpd* gene determined by qRT-PCR. Data were used to construct the Fig. 5

10.7717/peerj.6822/supp-12 Supplemental Information 12 Dataset S2. Relative expression of PNP biodegradation clusters genes determined by qRT-PCR, data were used for generation of Table 2.

## Additional Information and Declarations

### Data Availability

The following information was supplied regarding data availability: The supplemental data is available in the Figures, Tables, Tables S2–S10, and Datasets S1 and S2.

## References

[ref-1] Ahn J-H, Lee S-A, Kim S-J, You J, Han B-H, Weon H-Y, Lee S-W (2018). Biodegradation of organophosphorus insecticides with P-S bonds by two *Sphingobium* sp. strains. International Biodeterioration & Biodegradation.

[ref-2] Albers P, Weytjens B, De Mot R, Marchal K, Springael D (2018). Molecular processes underlying synergistic linuron mineralization in a triple‐species bacterial consortium biofilm revealed by differential transcriptomics. MicrobiologyOpen.

[ref-3] Allen KJ, Griffiths MW (2012). Impact of hydroxyl-and superoxide anion-based oxidative stress on logarithmic and stationary phase *Escherichia coli* O157:H7 stress and virulence gene expression. Food Microbiology.

[ref-4] Azaroff LS (1999). Biomarkers of exposure to organophosphorous insecticides among farmers’ families in rural El Salvador: factors associated with exposure. Environmental Research.

[ref-5] Bara JK, Soni R, Jaiswal S, Shrivastava K (2017). Review on bioremediation of methyl parathion contaminated agricultural soil by microorganisms. International Journal of Applied and Pure Science and Agriculture.

[ref-6] Barreto AC (2013). Quorum sensing: sistemas de comunicación bacteriana. Ciencia Actual.

[ref-7] Bech TB, Rosenbom AE, Sørensen SR, Jacobsen CS (2017). Conservative tracer bromide inhibits pesticide mineralisation in soil. Environmental Pollution.

[ref-8] Begum SS, Arundhati A (2016). A study of Bioremediation of Methyl Parathion in vitro using Potential Pseudomonas sp. isolated from Agricultural Soil, Visakhapatnam, India. International Journal of Current Microbiology and Applied Sciences.

[ref-9] Blunder S, Kõks S, Kõks G, Reimann E, Hackl H, Gruber R, Moosbrugger-Martinz V, Schmuth M, Dubrac S (2018). Enhanced expression of genes related to xenobiotic metabolism in the skin of patients with atopic dermatitis but not with ichthyosis vulgaris. Journal of Investigative Dermatology.

[ref-10] Briceño G, Schalchli H, Mutis A, Benimeli CS, Palma G, Tortella GR, Diez MC (2016). Use of pure and mixed culture of diazinon-degrading *Streptomyces* to remove other organophosphorus pesticides. International Biodeterioration & Biodegradation.

[ref-11] Brown NL, Stoyanov JV, Kidd SP, Hobman JL (2003). The MerR family of transcriptional regulators. FEMS Microbiology Reviews.

[ref-12] Cao K, Lai F, Zhao X-L, Wei Q-X, Miao X-Y, Ge R, He Q-Y, Sun X (2018). The mechanism of iron-compensation for manganese deficiency of *Streptococcus pneumoniae*. Journal of Proteomics.

[ref-13] Chakka D, Gudla R, Madikonda AK, Pandeeti EVP, Parthasarathy S, Nandavaram A, Siddavattam D (2015). The organophosphate degradation (*opd*) island borne esterase induced metabolic diversion in *Escherichia coli* and its influence on *p*-nitrophenol degradation. Journal of Biological Chemistry.

[ref-14] Chakrabarty AM (2017). Biodegradation and detoxification of environmental pollutants.

[ref-15] Chakraborty R, Wu CH, Hazen TC (2012). Systems biology approach to bioremediation. Current Opinion in Biotechnology.

[ref-16] Chauhan A, Pandey G, Sharma NK, Paul D, Pandey J, Jain RK (2010). *p*-Nitrophenol degradation via 4-nitrocatechol in *Burkholderia* sp. SJ98 and cloning of some of the lower pathway genes. Environmental Science & Technology.

[ref-17] Chen Q, Tu H, Luo X, Zhang B, Huang F, Li Z, Wang J, Shen W, Cui Z (2016). The regulation of *para*-nitrophenol degradation in *Pseudomonas putida* DLL-E4. PLOS ONE.

[ref-18] Cheng Y, Zang H, Wang H, Li D, Li C (2018). Global transcriptomic analysis of *Rhodococcus erythropolis* D310-1 in responding to chlorimuron-ethyl. Ecotoxicology and Environmental Safety.

[ref-71] Couoh-Uicab Y, Islas-Flores I, Canto-Canché BB (2010). Revisión de las características de los transportadores ABC involucrados en patogénesis fúngica. Tecnociencia, Chihuahua.

[ref-19] Davidson AL, Dassa E, Orelle C, Chen J (2008). Structure, function, and evolution of bacterial ATP-binding cassette systems. Microbiology and Molecular Biology Reviews.

[ref-20] De Lorenzo V (2008). Systems biology approaches to bioremediation. Current Opinion in Biotechnology.

[ref-21] Dennis P, Edwards EA, Liss SN, Fulthorpe R (2003). Monitoring gene expression in mixed microbial communities by using DNA microarrays. Applied and Environmental Microbiology.

[ref-22] De Waard MA, Andrade AC, Hayashi K, Schoonbeek H-J, Stergiopoulos I, Zwiers L-H (2006). Impact of fungal drug transporters on fungicide sensitivity, multidrug resistance and virulence. Pest Management Science.

[ref-23] Diagne M, Oturan N, Oturan MA (2007). Removal of methyl parathion from water by electrochemically generated Fenton’s reagent. Chemosphere.

[ref-24] Díaz E, Prieto MA (2000). Bacterial promoters triggering biodegradation of aromatic pollutants. Current Opinion in Biotechnology.

[ref-25] Dong ZC, Chen Y (2013). Transcriptomics: advances and approaches. Science China Life Sciences.

[ref-26] Dvořák P, Nikel PI, Damborský J, De Lorenzo V (2017). Bioremediation 3.0: engineering pollutant-removing bacteria in the times of systemic biology. Biotechnology Advances.

[ref-27] Egland PG, Harwood CS (1999). BadR, a new MarR family member, regulates anaerobic benzoate degradation by *Rhodopseudomonas palustris* in concert with AadR, an Fnr family member. Journal of Bacteriology.

[ref-28] Ekkhunnatham A, Jongsareejit B, Yamkunthong W, Wichitwechkarn J (2012). Purification and characterization of methyl parathion hydrolase from *Burkholderia cepacia* capable of degrading organophosphate insecticides. World Journal of Microbiology and Biotechnology.

[ref-29] Galperin MY, Makarova KS, Wolf YI, Koonin EV (2014). Expanded microbial genome coverage and improved protein family annotation in the COG database. Nucleic Acids Research.

[ref-30] Han Y, Gao S, Muegge K, Zhang W, Zhou B (2015). Advanced applications of RNA sequencing and challenges. Bioinformatics and Biology Insights.

[ref-31] Hernández-Mendoza A, Martínez-Ocampo F, Lozano-Aguirre Beltrán LF, Popoca-Ursino EC, Ortiz-Hernández L, Sánchez-Salinas E, Dantán-González E (2014). Draft genome sequence of the organophosphorus compound-degrading *Burkholderia zhejiangensis* strain CEIB S4-3. Genome Announcements.

[ref-32] Hoskisson PA, Rigali S (2009). Variation in form and function: the helix-turn-helix regulators of the GntR superfamily. Advances in Applied Microbiology.

[ref-33] Hrdlickova R, Toloue M, Tian B (2017). RNA‐seq methods for transcriptome analysis. Wiley Interdisciplinary Reviews: RNA.

[ref-34] Huang C-J, Wang Z-C, Huang H-Y, Huang H-D, Peng H-L (2013). YjcC, a c-di-GMP phosphodiesterase protein, regulates the oxidative stress response and virulence of *Klebsiella pneumoniae* CG43. PLOS ONE.

[ref-35] Jang H-J, Nde C, Toghrol F, Bentley WE (2008). Microarray analysis of toxicogenomic effects of ortho-phenylphenol in *Staphylococcus aureus*. BMC Genomics.

[ref-36] Kang Y, Norris MH, Zarzycki-Siek J, Nierman WC, Donachie SP, Hoang TT (2011). Transcript amplification from single bacterium for transcriptome analysis. Genome Research.

[ref-37] Kim S-J, Kweon O, Freeman JP, Jones RC, Adjei MD, Jhoo J-W, Edmondson RD, Cerniglia CE (2006). Molecular cloning and expression of genes encoding a novel dioxygenase involved in low-and high-molecular-weight polycyclic aromatic hydrocarbon degradation in *Mycobacterium vanbaalenii* PYR-1. Applied and Environmental Microbiology.

[ref-38] Krell T, Molina-Henares AJ, Ramos JL (2006). The IcIR family of transcriptional activators and repressors can be defined by a single profile. Protein Science.

[ref-39] Lazazzera BA (2000). Quorum sensing and starvation: signals for entry into stationary phase. Current Opinion in Microbiology.

[ref-40] Levin L, Carabajal M, Hofrichter M, Ullrich R (2016). Degradation of 4-nitrophenol by the white-rot polypore *Trametes versicolor*. International Biodeterioration & Biodegradation.

[ref-41] Li X, He J, Li S (2007). Isolation of a chlorpyrifos-degrading bacterium, *Sphingomonas* sp. strain Dsp-2, and cloning of the *mpd* gene. Research in Microbiology.

[ref-42] Li L, Zhou Z, Jin W, Wan Y, Lu W (2015). A transcriptomic analysis for identifying the unintended effects of introducing a heterologous glyphosate-tolerant EPSP synthase into *Escherichia coli*. Molecular BioSystems.

[ref-43] Liao H-Y, Chien C-C, Tang P, Chen C-C, Chen C-Y, Chen S-C (2018). The integrated analysis of transcriptome and proteome for exploring the biodegradation mechanism of 2,4,6-trinitrotoluene by *Citrobacter* sp. Journal of Hazardous Materials.

[ref-44] Liu J, Wang S, Qin T, Li N, Niu Y, Li D, Yuan Y, Geng H, Xiong L, Liu D (2015). Whole transcriptome analysis of *Penicillium digitatum* strains treatmented with prochloraz reveals their drug-resistant mechanisms. BMC Genomics.

[ref-45] Liu H, Zhang J-J, Wang S-J, Zhang X-E, Zhou N-Y (2005). Plasmid-borne catabolism of methyl parathion and *p*-nitrophenol in *Pseudomonas* sp. strain WBC-3. Biochemical and Biophysical Research Communications.

[ref-46] Livak KJ, Schmittgen TD (2001). Analysis of relative gene expression data using real-time quantitative PCR and the 2−ΔΔCT method. Methods.

[ref-47] Lowe R, Shirley N, Bleackley M, Dolan S, Shafee T (2017). Transcriptomics technologies. PLOS Computational Biology.

[ref-48] Luo F, Gitiafroz R, Devine CE, Gong Y, Hug LA, Raskin L, Edwards EA (2014). Metatranscriptome of an anaerobic benzene-degrading, nitrate-reducing enrichment culture reveals involvement of carboxylation in benzene ring activation. Applied and Environmental Microbiology.

[ref-49] McGettigan PA (2013). Transcriptomics in the RNA-Seq era. Current Opinion in Chemical Biology.

[ref-50] Min J, Wang B, Hu X (2017). Effect of inoculation of *Burkholderia* sp. strain SJ98 on bacterial community dynamics and *para*-nitrophenol, 3-methyl-4-nitrophenol, and 2-chloro-4-nitrophenol degradation in soil. Scientific Reports.

[ref-51] Mishra A, Khan J, Pandey AK (2017). Degradation of methyl parathion by a soil bacterial isolate: a pot study. Journal of Experimental Sciences.

[ref-52] Moreno-Medina DA, Sánchez-Salinas E, Ortiz-Hernández ML (2014). Removal of methyl parathion and coumaphos pesticides by a bacterial consortium immobilized in *Luffa cylindrica*. Revista Internacional De Contaminacion Ambiental.

[ref-53] Namouchi A, Gómez-Muñoz M, Frye SA, Moen LV, Rognes T, Tønjum T, Balasingham SV (2016). The *Mycobacterium tuberculosis* transcriptional landscape under genotoxic stress. BMC Genomics.

[ref-54] Nde CW, Jang H-J, Toghrol F, Bentley WE (2008). Toxicogenomic response of *Pseudomonas aeruginosa* to ortho-phenylphenol. BMC Genomics.

[ref-55] Pailan S, Gupta D, Apte S, Krishnamurthi S, Saha P (2015). Degradation of organophosphate insecticide by a novel *Bacillus aryabhattai* strain SanPS1, isolated from soil of agricultural field in Burdwan, West Bengal, India. International Biodeterioration & Biodegradation.

[ref-56] Pakala SB, Gorla P, Pinjari AB, Krovidi RK, Baru R, Yanamandra M, Merrick M, Siddavattam D (2007). Biodegradation of methyl parathion and *p*-nitrophenol: evidence for the presence of a *p*-nitrophenol 2-hydroxylase in a Gram-negative *Serratia* sp. strain DS001. Applied Microbiology and Biotechnology.

[ref-57] Pereira MA, Imada EL, Guedes RLM, Marchi FA, Cirillo PDR, Mateo EC (2017). RNA-Seq: applications and best practices. Applications of RNA-Seq and Omics Strategies-From Microorganisms to Human Health.

[ref-58] Perera IC, Grove A (2010). Molecular mechanisms of ligand-mediated attenuation of DNA binding by MarR family transcriptional regulators. Journal of Molecular Cell Biology.

[ref-59] Pope CN (1999). Organophosphorus pesticides: do they all have the same mechanism of toxicity?. Journal of Toxicology and Environmental Health, Part B.

[ref-60] Popoca-Ursino EC, Martínez-Ocampo F, Dantán-González E, Sánchez-Salinas E, Ortiz-Hernández ML (2017). Characterization of methyl parathion degradation by a *Burkholderia zhejiangensis* strain, CEIB S4-3, isolated from agricultural soils. Biodegradation.

[ref-61] Pratap SR, Manchanda G, Li ZF, Rai AR, Bhakta JN (2017). Insight of proteomics and genomics in environmental bioremediation. Handbook of Research on Inventive Bioremediation Techniques.

[ref-62] Romero-Rodríguez A, Robledo-Casados I, Sánchez S (2015). An overview on transcriptional regulators in Streptomyces. Biochimica et Biophysica Acta (BBA) - Gene Regulatory Mechanisms.

[ref-63] Serdar CM, Gibson DT, Munnecke DM, Lancaster JH (1982). Plasmid involvement in parathion hydrolysis by *Pseudomonas diminuta*. Applied and Environmental Microbiology.

[ref-64] Shen W, Liu W, Zhang J, Tao J, Deng H, Cao H, Cui Z (2010). Cloning and characterization of a gene cluster involved in the catabolism of *p*-nitrophenol from *Pseudomonas putida* DLL-E4. Bioresource Technology.

[ref-65] Sibanda S, Kwenda S, Tanui CK, Shyntum DY, Coutinho TA, Moleleki LN (2018). Transcriptome profiling reveals the EanI/R quorum sensing regulon in *pantoea ananatis* LMG 2665T. Genes.

[ref-66] Snoeck S, Greenhalgh R, Tirry L, Clark RM, Van Leeuwen T, Dermauw W (2017). The effect of insecticide synergist treatment on genome-wide gene expression in a polyphagous pest. Scientific Reports.

[ref-67] Srinivasan VB, Rajamohan G ((in press)). Genome analysis of urease positive *Serratia marcescens*, co-producing SRT-2 and AAC(6′)-Ic with multidrug efflux pumps for antimicrobial resistance. Genomics.

[ref-68] Thorvaldsdóttir H, Robinson JT, Mesirov JP (2013). Integrative genomics viewer (IGV): high-performance genomics data visualization and exploration. Briefings in Bioinformatics.

[ref-69] Tiwari B, Verma E, Chakraborty S, Srivastava AK, Mishra AK (2018). Tolerance strategies in cyanobacterium *Fischerella* sp. under pesticide stress and possible role of a carbohydrate-binding protein in the metabolism of methyl parathion (MP). International Biodeterioration & Biodegradation.

[ref-70] Tropel D, Van Der Meer JR (2004). Bacterial transcriptional regulators for degradation pathways of aromatic compounds. Microbiology and Molecular Biology Reviews.

[ref-72] Van Dyk JS, Pletschke B (2011). Review on the use of enzymes for the detection of organochlorine, organophosphate and carbamate pesticides in the environment. Chemosphere.

[ref-73] Vikram S, Pandey J, Bhalla N, Pandey G, Ghosh A, Khan F, Jain RK, Raghava GPS (2012). Branching of the *p*-nitrophenol (PNP) degradation pathway in *Burkholderia* sp. Strain SJ98: evidences from genetic characterization of PNP gene cluster. AMB Express.

[ref-74] Vikram S, Pandey J, Kumar S, Raghava GPS (2013). Genes involved in degradation of *para*-nitrophenol are differentially arranged in form of non-contiguous gene clusters in *Burkholderia* sp. strain SJ98. PLOS ONE.

[ref-75] Wang J, Chen L, Chen Z, Zhang W (2015). RNA-Seq based transcriptomic analysis of single bacterial cells. Integrative Biology.

[ref-76] Wang L, Chi X-Q, Zhang J-J, Sun D-L, Zhou N-Y (2014). Bioaugmentation of a methyl parathion contaminated soil with *Pseudomonas* sp. strain WBC-3. International Biodeterioration & Biodegradation.

[ref-77] Wang Z, Gerstein M, Snyder M (2009). RNA-Seq: a revolutionary tool for transcriptomics. Nature Reviews Genetics.

[ref-78] Wang J-P, Zhang W-M, Chao H-J, Zhou N-Y (2017). PnpM, a LysR-type transcriptional regulator activates the hydroquinone pathway in *para*-nitrophenol degradation in *Pseudomonas* sp. strain WBC-3. Frontiers in Microbiology.

[ref-79] Yan Y-W, Zou B, Zhu T, Hozzein WN, Quan Z-X (2017). Modified RNA-Seq method for microbial community and diversity analysis using rRNA in different types of environmental samples. PLOS ONE.

[ref-80] Yang JW, Zheng DJ, Cui BD, Yang M, Chen YZ (2016). RNA‐Seq transcriptome analysis of a *Pseudomonas* strain with diversified catalytic properties growth under different culture medium. MicrobiologyOpen.

[ref-81] Yang W, Zhou Y-F, Dai H-P, Bi L-J, Zhang Z-P, Zhang X-H, Leng Y, Zhang X-E (2008). Application of methyl parathion hydrolase (MPH) as a labeling enzyme. Analytical and Bioanalytical Chemistry.

[ref-82] Yong Y-C, Zhong J-J (2010). Recent advances in biodegradation in China: new microorganisms and pathways, biodegradation engineering, and bioenergy from pollutant biodegradation. Process Biochemistry.

[ref-83] Young MD, Wakefield MJ, Smyth GK, Oshlack A (2010). Gene ontology analysis for RNA-Seq: accounting for selection bias. Genome Biology.

[ref-84] Zhang J-J, Liu H, Xiao Y, Zhang X-E, Zhou N-Y (2009). Identification and characterization of catabolic *para*-nitrophenol 4-monooxygenase and *para*-benzoquinone reductase from *Pseudomonas* sp. strain WBC-3. Journal of Bacteriology.

[ref-85] Zhang S, Sun W, Xu L, Zheng X, Chu X, Tian J, Wu N, Fan Y (2012). Identification of the *para*-nitrophenol catabolic pathway, and characterization of three enzymes involved in the hydroquinone pathway, in *pseudomonas* sp. 1-7. BMC Microbiology.

[ref-86] Zhang T, Tang J, Sun J, Yu C, Liu Z, Chen J (2015). *Hex1*-related transcriptome of *Trichoderma atroviride* reveals expression patterns of ABC transporters associated with tolerance to dichlorvos. Biotechnology Letters.

[ref-87] Zhao G, Huang Q, Rong X, Cai P, Liang W, Dai K (2014). Biodegradation of methyl parathion in the presence of goethite: the effect of *Pseudomonas* sp. Z1 adhesion. International Biodeterioration & Biodegradation.

