# Supplementary material for: Transcriptional analysis reveals the metabolic state of Burkholderia zhejiangensis CEIB S4-3 during methyl parathion degradation
Source: PeerJ. 2019 Apr 24;7:e6822. doi: 10.7717/peerj.6822 (PMC6486813; doi:10.7717/peerj.6822)
Supplement: Supplemental Information 1 — Primers were designed based on to the nucleotide sequences of the selected genes. The methyl parathion hydrolase (mpd) gene, the genes that form p-nitrophenol catabolic clusters pnpABA′E1E2FDC and pnpE1E2FDC, and pnpG? gene that codifies for a possible p-nitrophenol monooxygenase-II. Recombinase A (recA) gene was used as an internal (unregulated) reference for relative quantification. [file peerj-07-6822-s001.docx]

**Table S1.** Primers used for gene validation by qRT-PCR.

| **Gene** | **Primers** |
| --- | --- |
| *recA* | *recA*f (5´-TAC AGC TAC AAC GGC GAT CG 3´)  *recA*r (5´-GCG ATT CGC GGA TCT TGT TC-3´) |
| *mpd* | *mpd*f (5´-TAC ATC ACC CAC ATG CAC CC-3´)  *mpd*r (5´-ATC GGC TTC TTT CTG GTC CG-3´) |
| *pnpC* | *pnpC*f (5´-AAA TCG TCG TTG GTG GTC GA-3´)  *pnpC*r (5´-CGC TTG ACG TCG AAA AAG GG-3´) |
| *pnpA* | *pnpA*f (5´-AGG AGG TGC GTC GTC GTA TG-3´)  *pnpA*r (5´-ATT CAG GTG GTC GAC GCG AT-3´) |
| *pnpA´* | *pnpA´*f (5´-TGA AAA GCT CAA CGG CGT GC-3´)  *pnpA´*r (5´-ACG AAG CGA CGT CAT TAC TCT-3´) |
| *pnpB* | *pnpB*f (5´-GCT ATC GCG CTG CAT TCG AA-3´)  *pnpB*r (5´-ACA TGT TGC CGA ATC GCG TC-3´) |
| *pnpC* | *pnpC*f (5´-AAA TCG TCG TTG GTG GTC GA-3´)  *pnpC*r (5´-CGC TTG ACG TCG AAA AAG GG-3´) |
| *pnpD* | *pnpD*f (5´-GCA TTG GCA TAC AAC CGC GA-3´)  *pnpD*r (5´-AAC AGC CTG CGG ACC ATC TT-3´) |
| *pnpE2* | *pnpE2*f (5´-GCA CTA CGC GAA GTC GAA CG-3´)  *pnpE2*r (5´-GGT TCT TCG CGA CAA CCA CC-3´) |
| *pnpE1* | *pnpE1*f (5´-CAT GGC GAC GTT CAA GGC AA-3´)  *pnpE1*r (5´-TCG CCA TGC TTG CTA CCG TA-3´) |
| *pnnF* | *pnnF*f (5´-GCA ATC GAG GCA AAT GCG GA-3´)  *pnnF*r (5´-ACG TCA AGA AAG CGC GTG TC-3´) |
| *pnpG?* | *pnpG?*f (5´-GCG AAG TCA TCA TGT GCG CA-3´)  *pnpG?r* (5´-TTT GCA AGG TCC ATC GAC GC-3´) |
